# CRISPR screen identifies the role of RBBP8 in mediating unfolded protein response induced liver damage through regulating protein synthesis

**DOI:** 10.1038/s41419-023-06046-x

**Published:** 2023-08-18

**Authors:** Heting Wang, Xuya Pan, Xiaoxin Xiang, Yang Zhang, Jianning Chen, Shiyi Wen, Jin Wang, Rong Gao, Jifeng Yang, Yaping Zhi, Siying Wen, Yubao Zheng, Ting Li, Heying Ai, Xuemin He, Yan Lu, Yanhua Zhu, Chunliang Li, Yanming Chen, Guojun Shi

**Affiliations:** 1 Department of Endocrinology and Metabolism, Medical Center for Comprehensive Weight Control, Guangdong Provincial Key Laboratory of Diabetology, Guangzhou Key Laboratory of Mechanistic and Translational Obesity Research, The Third Affiliated Hospital of Sun Yat-sen University, Guangzhou, China; 2 Department of Tumor Cell Biology, St. Jude Children’s Research Hospital, Memphis, USA; 3 Cancer Biology Program/Comprehensive Cancer Center, St. Jude Children’s Research Hospital, Memphis, USA; 4 Department of Pathology, The Third Affiliated Hospital of Sun Yat-sen University, Guangzhou, China; 5 Department of Infectious Diseases, The Third Affiliated Hospital of Sun Yat-sen University, Guangzhou, China; 6 Department of Clinical Immunology, The Third Affiliated Hospital of Sun Yat-sen University, Guangzhou, China; 7 State Key Laboratory of Oncology in Southern China, Sun Yat-sen University Cancer Center, Guangzhou, China

**Keywords:** Endoplasmic reticulum, Liver cancer

## Abstract

Unfolded protein response (UPR) maintains the endoplasmic reticulum (ER) homeostasis, survival, and physiological function of mammalian cells. However, how cells adapt to ER stress under physiological or disease settings remains largely unclear. Here by a genome-wide CRISPR screen, we identified that RBBP8, an endonuclease involved in DNA damage repair, is required for ATF4 activation under ER stress in vitro. RNA-seq analysis suggested that RBBP8 deletion led to impaired cell cycle progression, retarded proliferation, attenuated ATF4 activation, and reduced global protein synthesis under ER stress. Mouse tissue analysis revealed that RBBP8 was highly expressed in the liver, and its expression is responsive to ER stress by tunicamycin intraperitoneal injection. Hepatocytes with RBBP8 inhibition by adenovirus-mediated shRNA were resistant to tunicamycin (Tm)-induced liver damage, cell death, and ER stress response. To study the pathological role of RBBP8 in regulating ATF4 activity, we illustrated that both RBBP8 and ATF4 were highly expressed in liver cancer tissues compared with healthy controls and highly expressed in Ki67-positive proliferating cells within the tumors. Interestingly, overexpression of RBBP8 in vitro promoted ATF4 activation under ER stress, and RBBP8 expression showed a positive correlation with ATF4 expression in liver cancer tissues by co-immunostaining. Our findings provide new insights into the mechanism of how cells adapt to ER stress through the crosstalk between the nucleus and ER and how tumor cells survive under chemotherapy or other anticancer treatments, which suggests potential therapeutic strategies against liver disease by targeting DNA damage repair, UPR or protein synthesis.

## Introduction

Endoplasmic reticulum (ER) is an organelle responsible for synthesizing secretory proteins, including hormones, cytokines, carrier proteins, and apolipoproteins [1, 2]. ER protein homeostasis plays a critical role in maintaining cell function and survival, while its impairment is associated with various diseases [3, 4]. The secretory capacity of a mammalian cell is mainly dependent on ER homeostasis to adapt the physiological needs, while ER homeostasis is constantly challenged under physiological or pathological stimuli [2, 5, 6]. Thus, cells evolved a signaling pathway known as the unfolded protein response (UPR) to maintain protein homeostasis in the ER [7, 8]. Many ER proteins are misfolding prone as their maturation and folding require appropriate glycosylation and/or protein disulfide bond formation, while unfolded or misfolded proteins in the ER are potentially cytotoxic [8–10]. Thus, failure in the efficient and timely clearance of misfolded proteins leads to their accumulation in the ER that induces UPR, which is termed ER stress. Activation of ER stress aims to reduce misfolded protein and restore ER proteostasis [10, 11]. However, under pathological conditions, chronic or irremediable UPR induces cell death [4, 7, 12].

UPR is the master regulator of ER protein quality control machinery and is mediated by three ER-resident transmembrane proteins, PERK, IRE1α, and ATF6 [1]. Under steady-state, they are bound to BiP as inactivated forms [13, 14]. If misfolded proteins are not cleared in time, they will grab BiP away from the three UPR sensors, leading to the activation and the downstream signaling cascades that aim to release the stress by reducing protein synthesis, increasing ER-associated degradation (ERAD) capacity and ER volume, and increasing ER chaperon, respectively [13]. Among the three branches, the PERK pathway is the major regulator of protein synthesis in the ER. PERK phosphorylates eIF2α to reduce protein synthesis and release ER burden and activates ATF4, a transcription factor regulating protein translation, metabolism, oxidative stress, immune response, and cell survival [15, 16]. IRE1α activation leads to the activation of its RNase domain and splicing of XBP1u (unspliced) into XBP1s (spliced), which is a transcription factor controlling ER volume and many other genes involved in ER homeostasis [5]. Failure to adapt the ER stress contributes to the pathogenesis of multiple diseases, including obesity, type 2 diabetes, cancer, etc. In the meantime, the overactivation of UPR leads to cell death [4, 9, 16]. ATF4 is at the center of ER stress signaling and plays dual roles in cell fate decisions, depending on the severity and duration of the ER stress level. Under moderate and transient ER stress, ATF4 can be activated to use this ‘window of opportunity’ to promote the expression of adaptive genes [17]. However, irremediable or prolonged ER stress will result in chronic ATF4 activation with induction of genes leading to apoptosis, cell-cycle arrest, and senescence [18, 19]. Regarding the survival strategy, tumor cells also utilize the PERK–eIF2α–ATF4 pathway to reduce the stress resulting from rapid proliferation and nutrient limitation inside a growing tumor mass [17]. However, the detailed signaling mechanisms in regulating the dual role of ATF4 under various stress or physiological conditions remain unclear.

In this study, using a genome-wide CRISPR loss-of-function screen [20], coupled with an ATF4 reporter-based cell model, we identified and characterized the novel role of RBBP8, a DNA nuclease, in ATF4 activation. RBBP8 deficiency attenuated ATF4 activation in vitro with reduced protein synthesis and alleviated Tm-induced liver damage. Besides, RBBP8 was positively associated with ATF4 expression in liver cancer. This study reveals a novel role of RBBP8 in ATF4 activation that links DNA damage stress and UPR activation both in vitro and in vivo. It will provide insights into how cells adapt to various stresses and maintain survival through the crosstalk between the nucleus and ER under physiological and pathological conditions.

## Results

### Genome-wide CRISPR screen identifies a novel role of RBBP8 in ATF4 activation

**Fig. 1:**
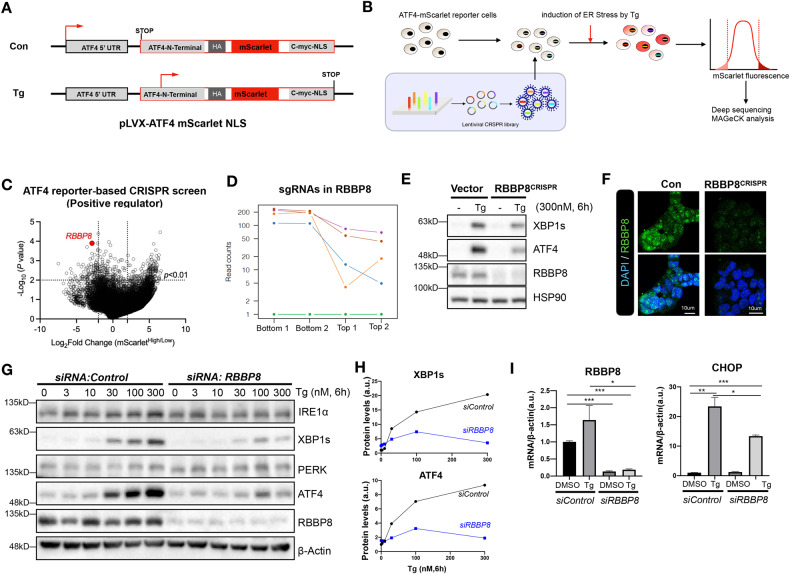
CRISPR screen targeting ATF4 reporter activity identifies RBBP8 as required for ATF4 activation. **A** Cartoon of the ATF4-mScarlet reporter construct. **B** Scheme of CRISPR screen. SEM leukemia cells with ATF4-mScarlet reporter were transfected with the genome-wide sgRNA CRISPR library, treated with ER stress inducer Tg (300 μM) for 24 h, and responders with lower or higher mScarlet fluorescence (top 10%) were sorted, followed by genomic DNA extraction and deep-sequencing. **C** The overall distribution of all sgRNAs from the screening was shown, and RBBP8 sgRNA was highlighted. **D** Five out of the six sgRNAs targeting RBBP8 from the library appeared in the sorted top and bottom fractions. Data were listed from two independent experiments. **E** Western blot analysis showing reduction of ATF4, XBP1s, and GADD34 in 293T cells under Tg treatment transduced with lentiviruses containing sgRNA targeting RBBP8. **F** Representative images of RBBP8 staining in HEK293T cells infected with lentivirus carrying CRISPR-Cas9 sgRNA against RBBP8. **G** Western blot analysis of RBBP8-deficient 293T cells by siRNA under Tg (300 nM) treatment for 6 h, and quantified as shown in (**H**). **I** The Q-PCR analysis of RBBP8 and CHOP mRNA in HEK293T cells transfected with siRNA against RBBP8 under Tg treatment. Data presented as mean ± SEM, *p < 0.05, **p < 0.01, and ***p < 0.001 by Student’s *t*-test. All data represent at least three independent experiments except those listed.

To explore the novel mechanisms underlying ATF4 activation, a lentivirus-based fluorescence reporter (mScarlet) construct under the control of the ATF4 gene promoter was employed in the SEM leukemia cell line, which is convenient for reporter-based gene screening [20]. Then reporter cells were infected by viruses bearing H3 sgRNA CRISPR library targeting over 18,000 genes with six sgRNAs each as described previously [20, 21]. As shown in Fig. 1A, B, cells were treated with ER stress inducer thapsigargin (Tg) for 24 h, and the top and bottom 10% of the virus-infected cells according to the mScarlet intensity were collected and subjected to deep sequencing, as shown in Fig. 1A, B [20]. The sequencing data were analyzed to identify sgRNAs and their corresponding target genes, followed by the scoring of the candidate ATF4 regulators by the MAGeCK program [22]. Through the analysis, 101 hits were identified as positive regulators (with lower ATF4 reporter activity) and 156 as negative regulators. As shown in Fig. 1C, D, retinoblastoma-binding protein 8 (RBBP8) stands out as a top candidate for ATF4 activation, and 4 of the 6 sgRNAs targeting RBBP8 were highly enriched at the bottom fraction compared with the top fraction. CRISPR KO of RBBP8 confirmed this result under Tg treatment in HEK293T cells (Fig. 1E). RBBP8 is an endonuclease functioning in DNA-end resection and is the first step of double-strand break (DSB) repair through the homologous recombination (HR) pathway [23, 24], and has emerged as a regulator of both cell cycle progression and repair of DNA double-strand breaks [25]. RBBP8 immuno-staining confirmed its nucleus distribution in HEK293T cells (Fig. 1F). To further validate the CRISPR screen result, RBBP8 was knocked down by siRNA in HEK293T cells. Both ATF4 and IRE1α downstream protein XBP1s were significantly induced by Tg treatment, while reduced with RBBP8 deletion (Fig. 1G, H). RBBP8 deletion efficiency and ATF4 inactivation marked by CHOP mRNA level were also confirmed by Q-PCR analysis (Fig. 1I). These data indicate that RBBP8 plays an essential role in ATF4 activation under ER stress in vitro.

### RBBP8 maintains cell cycle progression and protein translation

**Fig. 2:**
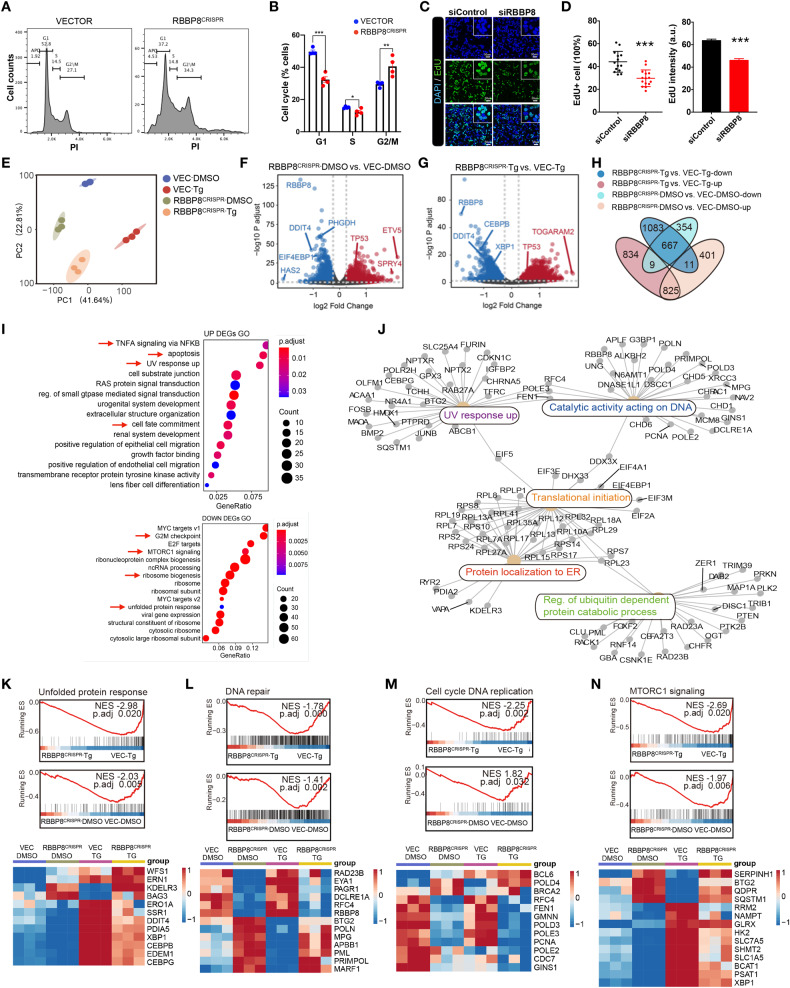
Transcriptomic analysis of RBBP8-deficient HEK293T cells under basal or ER stress conditions. **A** Cell cycle analysis of HEK293T cells 48 h post lentivirus infection. Histograms show the patterns of cells stained with PI. Cells were divided into Apoptotic (APO), G1, S, and G2/M fractions and quantified in (**B**). **C** HEK293T cells were transfected with siRNAs and incubated with EdU for 2 h. Fluorescence images were used to detect EdU and quantified in (**D**). **E** Principal component analysis (PCA) plot of transcriptomic analysis for HEK293T cells with control (DMSO), TG treatment (300 nM for 6 h) of either vector or RBBP8^CRISPR^ knockout cells. *n* = 3 for each group. **F**, **G** Volcano plots for differentially expressed genes (DEGs) of RBBP8-KO vs. VEC with DMSO control (**F**) and under Tg treatment (**G**). DEGs are selected by *P* < 0.05 and |log2 (fold change) |>0.25. Significantly up- and down-regulated genes are represented as red and gray dots. **H** Venn diagram represents the overlap of DEGs identified by RNA-sequencing data. **I** Gene ontology (GO) enrichment analysis of DEGs that significantly changed under DMSO and Tg treatment from RBBP8-KO vs. Control cells indicated in (**H**). **J** Cnet plot for enriched pathway genes of overlap DEGs that changed with DMSO and Tg treatment. From RBBP8-KO vs. Control cells. **K–N** Gene set enrichment analysis (GSEA) enrichment and heatmap analysis showed the row-scaled gene expression of “Unfolded protein response” (**K**), “DNA repair” (**L**), “Cell cycle DNA replication” (**M**), “MTORC1 signaling” (**N**) pathways in RBBP8-KO 293T cells with TG or vehicle-treated groups compared to respective Vector groups. All data represent at least three independent experiments. Data presented as mean ± SEM, *p < 0.05, **p < 0.01, and ***p < 0.001 by Student’s *t*-test.

RBBP8 is involved in cell cycle progression during the S/G2 phase and growth retardation [24]. Consistently, HEK293T cells with RBBP8 deletion also showed impaired cell cycle progression demonstrated by PI staining (Fig. 2A, B), EdU staining (C-D), and retarded growth (Supplementary Fig. S1A–C). Thus, we used a mixture of infected cells with CRISPR-mediated RBBP8 deficiency for the following analysis. However, how the nucleus protein RBBP8 regulates PERK-ATF4 activation remains unclear. Interestingly, RBBP8-deficient cells seem to be more resistant to Tg-induced cell viability (Supplementary Fig. S1D). To explore how RBBP8 is involved in ATF4 activation, RBBP8-deficient HEK293T cells were subjected to RNA sequencing. Bioinformatic analysis showed that RBBP8 deficiency led to transcriptomic change under basal or ER stress conditions (Fig. 2E). Volcano plots indicated genes that were significantly changed with RBBP8 deficiency under control (Fig. 2F) or Tg treatment (Fig. 2G). These genes were divided by up or down-regulation, and overlapped genes were analyzed by the Venn diagram, as shown in Fig. 2H. Then the overlapped (1512) genes significantly changed with RBBP8 deficiency regardless of Tg treatment and were pooled together and subjected to pathway analysis, while RBBP8-deficient cells at basal condition were analyzed separately (Supplementary Fig. S1E). Interestingly, genes involved in DNA damage response were up-regulated, while cell cycle, protein synthesis, and UPR-related genes were down-regulated with RBBP8 deficiency (Fig. 2I), which are further analyzed with cnet plot to visualize functional enrichment of genes (Fig. 2J). Detailed gene expression changes were specifically listed (Fig. 2K–N). As the reduction of protein synthesis is a critical mechanism in attenuating ER stress, it is proposed that RBBP8 deficiency led to impaired DNA damage repair and cell cycle progression, resulting in decreased protein synthesis and further attenuated ER stress response.

**Fig. 3:**
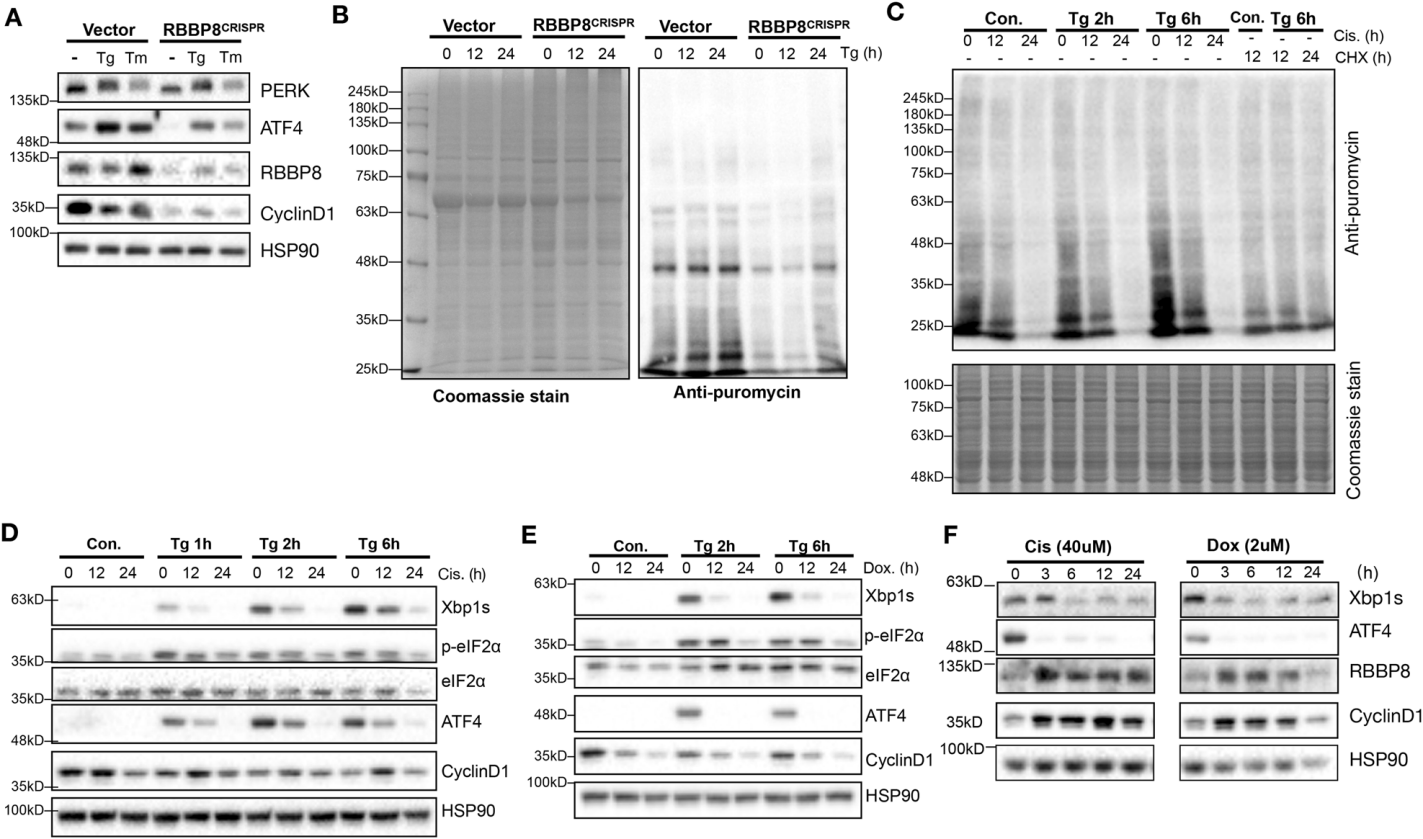
RBBP8 deficiency or treatment by genotoxic agents leads to attenuated UPR and reduced protein synthesis. **A** Western blot analysis of RBBP8-deficient HEK293T cells generated through the CRISPR/Cas9 system under Tg (300 nM) or Tm treatment (2.5 μg/ml). **B** WT and RBBP8-deficient HEK293T cells were pulse-labeled with puromycin after Tg (300 nM) treatment for the indicated time. **C** HepG2 cells pretreated with cisplatin (Cis, 40 μM) and cycloheximide (CHX, 10 μg/ml) were treated with control or Tg treatment for the indicated time, followed by pulse-labeling with puromycin. Cell lysates after puromycin labeling were subjected to western blot analysis and Coomassie blue staining was used as the loading control. **D**, **E** Western blot analysis of protein expression in HEK293T cells pretreated with cisplatin or doxorubicin (Dox), respectively, for 0, 12, and 24 h, followed by Tg treatment for the indicated time. **F** Western blot protein expression analysis in HEK293T cells treated with cisplatin (40 μM) or doxorubicin (2 μM) for the indicated time. All data represent at least three independent experiments.

To test the hypothesis above, the protein synthesis capacity of WT and RBBP8-deficient cells was analyzed by labeling with puromycin (Supplementary Fig. S2A, B) [23, 26]. Expression levels of ATF4, RBBP8, and cell cycle-regulated gene CyclinD1 were confirmed in HEK293T cells with RBBP8 deficiency (Fig. 3A). At the same time, puromycin-labeled newly synthesized proteins were decreased (Fig. 3B). As genotoxic drugs induce DNA damage response, cell cycle arrest, and reduce cell proliferation, which demonstrated similar phenotype with RBBP8 deficiency [26], we proposed that genotoxic drug could also attenuate unfolded protein response by inducing DNA damage and reducing global protein synthesis. Indeed, cisplatin treatment reduced newly synthesized protein (Fig. 3C). Further analysis showed that either Cisplatin or Doxorubicin treatment showed impaired CyclinD1 expression and reduced protein levels of Xbp1s, ATF4, and eIF2a phosphorylation, either under Tg treatment (Fig. 3D, E) or control conditions (Fig. 3F). These data support the hypothesis that both RBBP8 deficiency and treatment by genotoxic drugs led to DNA damage response, cell cycle arrest and protein synthesis reduction, which may further attenuate UPR under ER stress.

### RBBP8 is highly expressed in mouse liver and its expression is responsive to ER stress

**Fig. 4:**
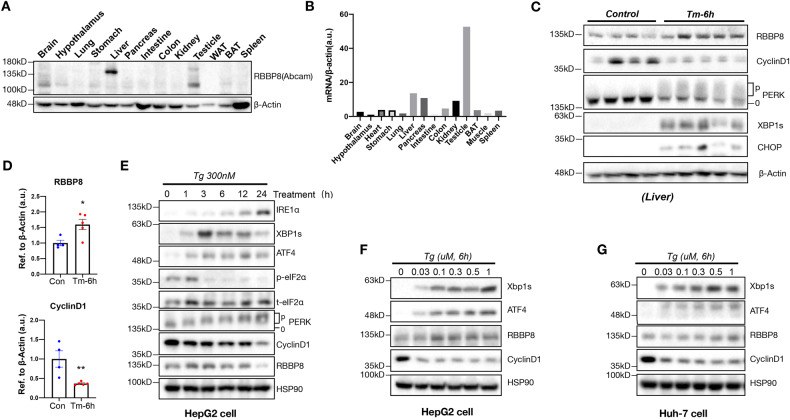
RBBP8 is highly expressed in the liver, and its expression is regulated by ER stress in vitro and in vivo. **A** Protein expression of RBBP8 in various tissues from C57BL/6 mice by Western blot analysis. **B** The mRNA expression of RBBP8 in various tissues from C57BL/6 mice by Q-PCR. **C**, **D** Western blot protein expression analysis in Tunicamycin injection (1 mg/kg body weight, i.p.) or control livers from mice (8 weeks). PERK phosphorylation was analyzed by Phos-tag PAGE gel and RBBP8 and CyclinD1 protein levels were quantified in (**D**). **E**, **F** Western blot protein expression analysis in HepG2 cells treated with Tg at the indicated time (**E**) or the indicated dose (**F**). **G** Western blot protein expression analysis of UPR marker, RBBP8, and CyclinD1 expression in Huh-7 cells treated with Tg at the indicated dose. All data represent at least three independent experiments. Data presented as mean ± SEM, *p < 0.05, **p < 0.01 by Student’s *t*-test.

To explore the physiological role of RBBP8, its expression pattern was analyzed in various mouse tissues. As shown in Fig. 4A, B, the liver and testis are among the tissues with relatively high expression at protein and mRNA levels. UPR and DNA damage response are two critical pathways in regulating proteome homeostasis and genome integrity, which are shown to interplay via multiple mechanisms [51]. Thus, RBBP8 expression was analyzed under ER stress with an intraperitoneal injection of tunicamycin in mice (Fig. 4C, D), showing that the cell cycle was impaired and RBBP8 expression was upregulated under acute ER stress. Interestingly, RBBP8 expression decreased with time in the liver and relieved ER stress response (Supplementary Fig. S3A, B), suggesting that RBBP8 expression is associated with ER stress response. Further, RBBP8 expression was confirmed under Tg or Tm treatment in HCC cell lines (Fig. 4E–G), confirming the dynamic expression in response to ER stress. These data showed that nucleus-localized protein RBBP8 is responsive to ER stress in various cell types in a time and cell-type-specific manner, suggesting a conserved role of RBBP8 in linking DNA damage response and UPR.

### RBBP8 deficient hepatocytes are resistant to ER stress-induced damage

**Fig. 5:**
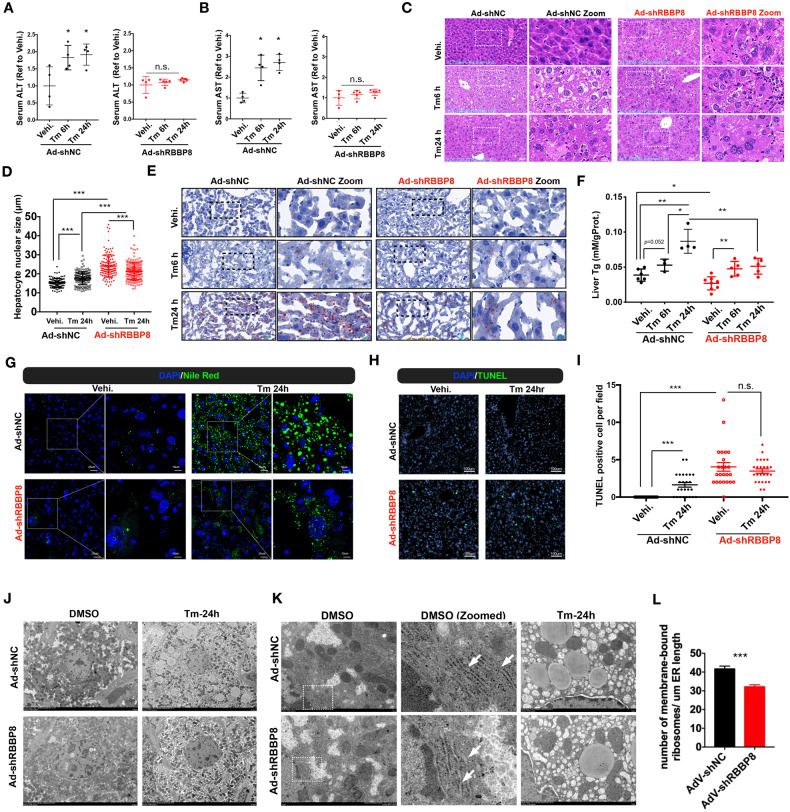
RBBP8-deficient hepatocytes were resistant to Tm-induced liver injury. Male C57BL/6 J mice aged 16–20 weeks were intravenously injected with Ad-shNC or Ad-shRBBP8. 16 days post virus injection, mice were intraperitoneally injected with Tm (1 mg/kg, i.p.) for 6 or 24 h, or with the vehicle for 24 h. Serum ALT levels (**A**) and AST levels (**B**) of Ad-shNC and Ad-shRBBP8 mice (right) normalized with respective control mice injected with the vehicle. *n* = 4–5 for each group. **C**, **D** Representative H&E staining of liver sections, showing hepatocyte ballooning (arrows) and necrosis (asterisk) after Tm injection, and vacuolar degeneration in nuclei (triangle) after RBBP8 knockdown. Nucleus diameters from H&E staining were quantified in (**D**). *n* = 4–5 for each group. **E** Representative Oil Red O staining of liver sections from mice as described above. *n* = 4–5 for each group. **F** Triglyceride (Tg) contents were extracted and quantified from the livers of represented groups. **G** Representative Nile Red fluorescent microscopic analysis of liver sections from mice described above. **H**, **I** TUNEL staining of liver sections and the quantification of TUNEL-positive hepatocytes. Data presented as mean ± SEM, ***p < 0.001. *n* = 4–5 mice for each group. **J–L** TEM analysis of liver sections from control or Tunicamycin (1 mg/kg) treated mice, showing the structure of nucleus and ER (**J**), ER-ribosome localization with zoomed images and lipid droplets (**K**), and quantification of ER membrane-bound ribosome intensity (**L**). *n* = 2 mice for each group. Data presented as mean ± SEM, *p < 0.05, **p < 0.001, ***p < 0.001 by Student’s *t*-test.

To further examine the physiological role of RBBP8 in the liver, we generated hepatic RBBP8-deficient mice by adenovirus-mediated shRNA. First, the deletion efficiency of siRNA and the corresponding sequence with adenovirus-mediated shRNA were confirmed in MEF and Hepa1-6 (Supplementary Fig. S3C–H), as well as the role of RBBP8 in ATF4 activation in mouse cells. Then, RBBP8 was reduced by intravenous injection of adenovirus carrying shRBBP8 tested above, followed by control or tunicamycin injection. RBBP8 deletion efficiency was confirmed at 17 days post-virus injection (Supplementary Fig. S5A–C). Mice with RBBP8 deficiency showed resistance to Tm-induced hepatocyte injury as indicated by serum ALT and AST activity (Fig. 5A, B), while it is not surprising that RBBP8 deficiency also leads to elevated ALT and AST levels due to its critical function as described above (Supplementary Fig. S4A, B). Also, H&E staining showed that RBBP8 deficiency in hepatocytes promoted resistance to further injury by Tm (Fig. 5C, D). As liver injury induced by tunicamycin is represented by lipid accumulation in hepatocytes, liver sections were stained with Oil Red O (Fig. 5E) and Nile Red (Fig. 5G), as well as biochemical quantification of the liver lysates (Fig. 5F). These data indicated that RBBP8 deficiency showed less induction of lipid accumulation after tunicamycin injection. TUNEL staining also confirms the resistance of RBBP8-deficient hepatocytes to ER stress exaggerated cell death (Fig. 5H, I). Ultrastructure of hepatocytes by transmission electronic microscope (TEM) demonstrated the expanded nucleus volume with RBBP8 deficiency (Fig. 5I, 5D) and fewer lipid droplets after tunicamycin treatment for 24 h (Fig. 5K, E–G). ER morphology indicated less ribosome localization on the ER outer membrane in RBBP8-deficient hepatocytes (Fig. 5K, L). In summary, these data suggest that RBBP8-deficient hepatocytes are resistant to tunicamycin exaggerated liver injury and cell death.

**Fig. 6:**
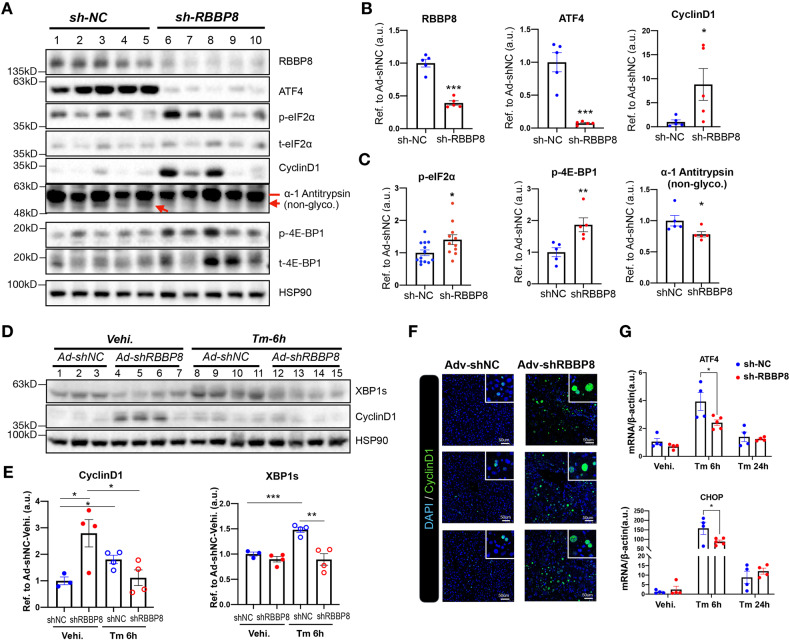
RBBP8-deficient hepatocytes showed attenuated ATF4 activation in the liver. **A–C** Western blot analysis of hepatic protein levels in Ad-shNC and Ad-shRBBP8 treated mice 3 days post virus injection (**A**), with quantification shown in **B** and **C**, refer to HSP90 except stated. *n* = 4–5 for each group, except for p-eIF2α were combined with two independent experiments. **D**, **E** Western blot analysis of hepatic protein levels in mice sixteen days post virus injection (14–15 weeks, male) followed by Tm injection (1 mg/kg i.p.) for the indicated time (**D**), with quantification of XBP1s and CyclinD1 protein level in (**E**). *n* = 3–4 for each group. **F**, **G** Representative immunofluorescence images for CyclinD1 staining in mice liver sections (**F**) and mRNA levels of ATF4 and CHOP in mice liver tissues (**G**) were analyzed 17 days post virus injection, followed by Tm treatment or vehicle control. *n* = 4–5 for each group. Data presented as mean ± SEM, *p < 0.05, **p < 0.01, ***p < 0.001, Student’s *t*-test.

To explore the signaling mechanism of RBBP8 in liver injury with acute RBBP8 deficiency, UPR activation was examined 3 days post adenovirus injection. As shown in Fig. 6A–C, with the successful reduction of RBBP8 protein, both RBBP8 and ATF4 expression was significantly reduced, with impaired cell cycle progression (CyclinD1) and decreased protein synthesis (p-4E-BP1 and p-eIF2α). To further study the role of RBBP8 in ER homeostasis, sucrose gradient ultracentrifuge analysis was performed with liver lysates, showing the alteration of ER protein homeostasis with RBBP8 deficiency (Supplementary Fig. S6A–D). Day 17 post virus injection of Adv-shRBBP8, XBP1s protein levels from liver tissues were also considerably reduced, showing reduced ER stress response at basal and ER stress conditions, while induced CyclinD1 was applied as a marker of RBBP8 deficiency (Fig. 6D–F). mRNA level of ATF4 and CHOP was significantly reduced under Tm treatment (Fig. 6G). These data demonstrated that RBBP8 was essential in maintaining cell cycle progression, and its deficiency led to cell cycle arrest, attenuated protein synthesis, alleviated ER stress response, and improved resistance to ER stress-induced injury in hepatocytes.

### RBBP8 and ATF4 expressions are elevated in liver cancer

**Fig. 7:**
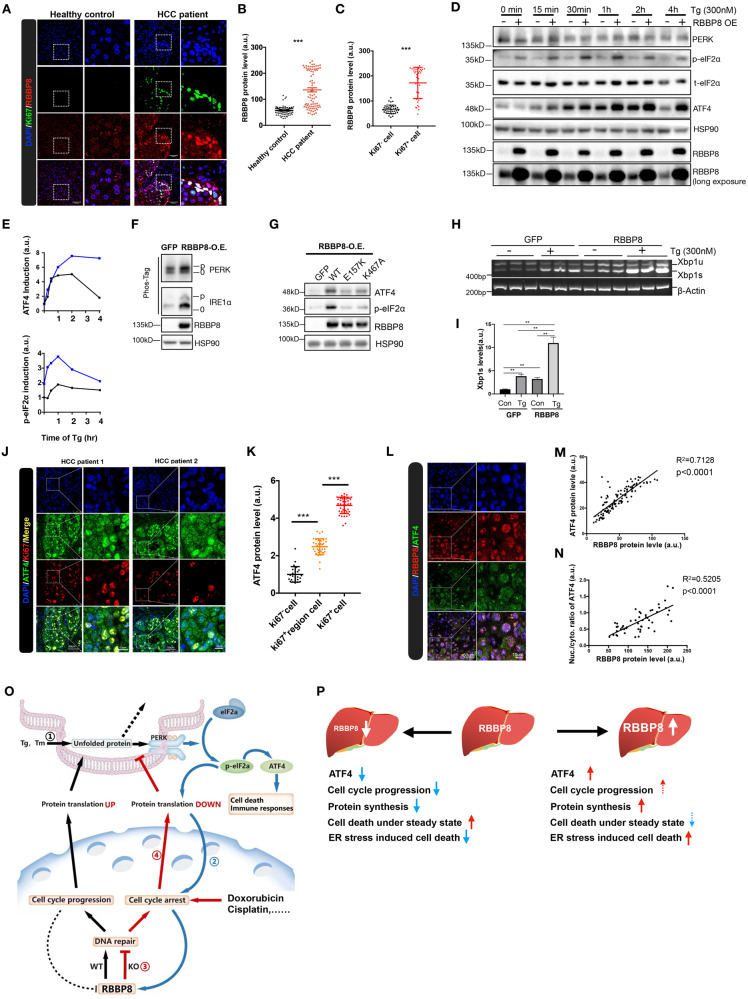
Increased expression of RBBP8 and ATF4 in liver cancer. **A** Representative images of RBBP8 (red) and Ki67 (green) co-staining in liver samples from patients with or without hepatocellular carcinoma (HCC), with quantification of RBBP8 intensity in **B**, and quantification of RBBP8 intensity of cancer patients divided by Ki67+ or Ki67− cells shown in (**C**). Each data point in **B** and **C** represent one field of observation. *n* = 4 for patient with HCC, *n* = 2 for non-HCC patients. **D** Western blot analysis for UPR pathway in HEK293T cells transfected with RBBP8 plasmid under Tg (300 nM) treatment for the indicated time. Quantification of protein expression levels of ATF4 and p-eIF2α was shown in (**E**). Data represents at least two independent experiments. **F** Activation of IRE1α and PERK by Phos-tag assay in HEK293T cells with RBBP8 or GFP over-expression. Data represent at least two independent experiments. **G** Western blot analysis of ATF4 activation in HEK293T cells with WT or mutant RBBP8 (E157K, S467A) over-expression. Data represent at least three independent experiments. **H** RT-PCR for Xbp1 splicing in HEK293T cells over-expressed with WT RBBP8 after Tg treatment, and Xbp1s band intensity was quantified in (**I**). Data represent at least two independent experiments. **J** Representative immunofluorescence staining of ATF4 (red) and Ki67 (green) in the liver sections from HCC patients and quantification was shown in (**K**). Each dot represents one field of observation. *n* = 3 for HCC patients. **L** Representative immunofluorescent staining of RBBP8 (red) and ATF4 (green) in the liver sections from HCC patients. Correlation analysis for immunofluorescence intensity of ATF4 and RBBP8 (**M**) and the nucleus to cytosol ratio of ATF4 and RBBP8 (**N**) in HCC patients. Each dot represents one field observed. *n* = 3 for HCC patients. Data were analyzed by linear regression, and *p* < 0.0001 was represented for significant correlation. Data presented as mean ± SEM, *p < 0.05, **p < 0.01,***p < 0.001, Student’s *t*-test. **O**, **P** A schematic model proposing how RBBP8 is involved in ATF4 activation in vitro and in vivo. **O** ER stress activates the PERK-ATF4 pathway and inhibits protein synthesis, leading to DNA damage response and cell cycle arrest with induced RBBP8 expression. RBBP8 deficiency or treatment by genotoxic drugs leads to DNA damage repair deficiency and cell cycle arrest, which reduces cell proliferation and induces cell death. In the meantime, DNA damage response also inhibited global protein synthesis, decreased misfolded protein accumulation in the ER, and attenuated UPR activation. **P** Physiologically, RBBP8 deficiency in hepatocytes leads to increased cell death but promotes resistance to Tm-induced cell death and liver injury with reduced ATF4 activation. In clinical samples, RBBP8 is elevated in HCC patients and associated with ATF4-induced expression and ATF4 nucleus localization. With dashed arrows indicating that more direct evidence will be needed to support the conclusion, our model suggests that increased RBBP8 might lead to increased DNA damage repair capacity associated with adaptation capacity to ER stress through activating ATF4, which might promote tumorigenesis.

DNA damage response and UPR play critical roles in tumorigenesis [2]. Liver injury and impaired lipid accumulation are liver cancer risk factors [27–29]. Thus, liver cancer provides a model to study the physiological roles of RBBP8 and ATF4 in human disease. As analyzed from the TCGA database, RBBP8 mRNA expression is significantly increased in multiple cancers, including hepatocellular liver carcinoma (LIHC) [30], and its high expression is correlated with poor survival (Supplementary Fig. S7A, B). Thus, we analyzed the protein expression in liver cancer patients and healthy donors. Consistently, Ki67-positive regions (tumor) showed significantly higher RBBP8 expression than the negative regions, and Ki67-positive cells (proliferating cells) showed significantly higher RBBP8 expression than negative cells, as shown in Fig. 7A–C. To analyze the role of increased RBBP8 expression in ATF4 activation, HEK293T cells were overexpressed with RBBP8 and analyzed with UPR signaling followed by Tg treatment. Ectopic RBBP8 expression increased PERK activation indicated by increased ATF4 protein level and eIF2α phosphorylation (Fig. 7D, E). Interestingly, the overexpression of RBBP8 alone also activated PERK and IRE1α as indicated by their phosphorylation analysis of the Phos-tag gel (Fig. 7F) [31]. Mutations (E157K or K467A) that abolish RBBP8 function in cell cycle progression showed reduced ATF4 and p-eIF2α protein levels (Fig. 7G) [32, 33]. IRE1α activation was also increased, indicated by spliced Xbp1 mRNA level (Fig. 7H, I).

To further explore the involvement of RBBP8 expression in ATF4 activation under disease setting, ATF4 mRNA expression was also analyzed in the TCGA database. Interestingly, ATF4 mRNA was highly expressed in various tumors, including liver cancer, and its expression level was negatively correlated with patient survival (Supplementary Fig. S7C, D). Further, in situ, RBBP8 and ATF4 expression levels were analyzed by co-immunostaining in tumor samples from hepatocellular carcinoma patients. Firstly, the effectiveness of the ATF4 antibody was validated by human endothelial cells freshly transfected with the ATF4-CRISPR construct (Supplementary Fig. S5D), and ATF4 staining efficiency was confirmed by ectopic expression of Flag-tagged ATF4 (human) in AML12 mouse hepatocytes (Supplementary Fig. S5E). As expected, ATF4 expression was mainly localized in the nucleus in vitro. These data demonstrated that both endogenous human and mouse ATF4 proteins were recognized by the ATF4 antibody used in this study through Western blot and immunostaining. Then ATF4 expression in liver cancer was analyzed by immunostaining, showing that ATF4 protein expression was significantly higher in Ki67-positive region and Ki67-positive cells (Fig. 7J, K). These data suggest that highly proliferative cells and tumor cells require higher UPR signaling, and ATF4 might be an essential gene for liver tumorigenesis. Interestingly, when RBBP8 and ATF4 were costained in liver cancer samples, they showed a positive correlation throughout the tumor sections (Fig. 7L, M), and the ratio of ATF4 nucleus localization is also positively correlated with RBBP8 protein level (Fig. 7N), suggesting the involvement of RBBP8 in ATF4 signaling in human cancer cells both in vitro and in vivo. RBBP8 expression was also analyzed in multiple liver cancer cell lines at basal or cell cycle synchronized cells, showing that RBBP8 was positively associated with ATF4 expression at the protein level (Supplementary Fig. S9A–C). Besides, loss- and gain-of-function of RBBP8 in ATF4 activation were also confirmed in HCC cell lines in vitro (Supplementary Fig. S9D–H), which indicated that elevated RBBP8 expression promoted ATF4 activation dependent on increasing protein synthesis.

These data suggest that RBBP8 is required for ATF4 activation both in vitro and in vivo. RBBP8 or ATF4 protein expression were potentially diagnostic markers and therapeutic targets of liver cancer.

## Discussion

UPR is an essential mechanism in maintaining or restoring proteostasis and functionality of ER. However, prolonged or irremediable UPR leads to cell death [14]. While the signaling pathways activating UPR were well characterized, how UPR signaling is deactivated to limit its severity and duration to avoid cell death remains unclear [19, 28]. Using a CRISPR-based loss-of-function genetic screen, we illustrated that cells with RBBP8 deficiency alleviated ATF4 activation under ER stress, and further explored the mechanism of how RBBP8 was involved in ATF4 activation in vitro and in vivo. Our study reveals a novel regulatory mechanism in UPR signaling that cells deficient with DNA damage repair gene(s) could be resistant to UPR activation under ER stress, providing novel insights into diagnosis and therapeutics against tissue injury and cancer.

Our results show that RBBP8 deficiency leads to cell cycle arrest and retarded cell proliferation while alleviating ATF4 activation (Figs. 1, 2) [34, 35]. RNA-seq analysis demonstrates that RBBP8 deficiency alters cell cycle progression, protein translation, and ER proteostasis (Fig. 3), which suggests that cells with RBBP8 deficiency attenuated UPR activation under ER stress by reducing protein synthesis. Cisplatin and doxorubicin are genotoxic drugs that potentially mimic RBBP8 deficiency, and their treatment showed reduced protein synthesis and resistance to UPR activation under control or ER stress conditions (Fig. 3). These data demonstrate that impaired DNA damage repair induced by gene deficiency or drug treatment attenuates ER stress response through reducing global protein synthesis. Also, these results suggest that cancer cells, when treated with genotoxic drugs, may go through cell death or cell cycle arrest; however, if they survived, they might be more tolerant to ER stress and potentially other stresses, thus showing proliferation advantage.

Tissue distribution analysis suggests the involvement of RBBP8 in liver physiology (Fig. 4). Interestingly, as it was reported that UPR activation also inhibited cell cycle progression [6, 16], RBBP8 protein expression was dynamically changed under ER stress both in vitro and in vivo, suggesting a possible regulatory loop between DNA damage response and UPR activation. Mice with RBBP8 deficiency showed resistance to ALT and AST induction by tunicamycin, alleviated further hepatocyte injury, reduced lipid accumulation induction after tunicamycin injection, and were more resistant to ER stress-induced apoptosis (Fig. 5). Further biochemical analysis of liver samples confirmed that RBBP8-deficient hepatocytes showed attenuated UPR response (Fig. 6). Our data suggested that elevated RBBP8 expression led to ATF4 activation through increasing protein synthesis (Supplementary Figs. S8 and S9), while the detailed mechanism remains to be illustrated. These data suggest that RBBP8 deficiency could protect cells from further injury under ER stress.

Tumor cells undergo a high proliferative rate that requires a large amount of DNA and protein synthesis, associated with more DNA damage and UPR activation. TCGA database showed that both RBBP8 and ATF4 mRNA levels were highly expressed in multiple cancer types, including liver cancer, and demonstrated its association with a shorter life span in HCC patients (Supplementary Fig. S7A–D), and RBBP8 expression was highly expressed in either Ki67-positive regions or positive cells. PERK-ATF4 and IRE1α-Xbp1s UPR pathways were hyperactivated under basal or ER stress conditions in cells overexpressed with RBBP8 in vitro (Fig. 7). We confirmed that both RBBP8 and ATF4 were highly expressed in the Ki67-positive regions and positive cells in HCC tissues (Fig. 7), and discovered that RBBP8 protein expression level was significantly correlated with ATF4, supporting the hypothesis that RBBP8 is required for ATF4 activation and contributes to the progression of liver cancer. It is noteworthy to mention that unlike previous reports indicating ATF4 as an inducible transcription factor, our data strongly suggested that ATF4 protein was highly expressed in both normal tissue and tumor in the liver; however, the ratio of ATF4 in nucleus vs. cytosol, which indicated its transcriptionally active form, was increased both in hepatocytes with Tm treatment and in Ki67-positive tumor cells (Fig. 7). These findings provide the diagnostic potential that increased RBBP protein expression and ATF4 expression, as well as its nucleus distribution, may be associated with the more proliferative capacity of tumor cells and worse outcomes in HCC patients. Also, developing drugs targeting RBBP8 or ATF4 might be a novel therapeutic strategy against HCC.

In summary, using a CRISPR-based genetic screen, we uncovered RBBP8 as a novel mediator of ER stress-induced ATF4 activation. As illustrated in Fig. 7, we propose that RBBP8 is required for ATF4 activation under ER stress through regulating DNA damage response and protein synthesis. In mouse models, RBBP8 deficiency in hepatocytes attenuated ATF4 activation and ER stress-induced cell death. In HCC patients, RBBP8 was elevated and positively associated with ATF4 expression and activity (shown by nucleus localization), indicating the critical role of RBBP8 in ATF4 activation. Further characterizing the role of RBBP8 and ATF4 in HCC pathogenesis by various transgenic mouse models will be helpful in developing therapeutics against HCC or other cancer types. Thus, this study provides a new clue in understanding the crosstalk mechanisms between the nucleus and ER and the pathogenesis of liver cancer.

## Materials and methods

### Cell culture and treatment

SEM cells (ACC-546, DSMZ), HepG2, and Hepa1–6 cells (kindly provided by Dr. Mingqiang Li, Sun Yat-sen University, Guangzhou, China), Huh-6, Huh-7, SNU-387 and RBE cells (kindly provided by SequMed Biotech Inc., Guangzhou, China) were cultured as described in Supporting Experimental Procedure.

### CRISPR screening

The genome-scale human CRISPR KO H3 library (Addgene #133914) and pLVX-ATF4 mScarlet NLS reporter plasmid (Addgene #115969) were purchased from Addgene. The reporter constructed was packaged into lentivirus, and further infected by the pooled H3 sgRNA library at low M.O.I (~0.3), followed by sorting for mScarlet^High^ and mScarlet^Low^ populations at day 7 post-infection. The sequencing and combined analysis of sgRNAs against each human gene was conducted using the MAGeCK algorithm [36], and details were described in the Supporting Experimental Procedure.

Western blot, siRNA, shRNA and overexpression of target genes, histological analysis and Immunofluorescence staining and quantification, cell cycle and cell viability analysis, quantitative and reverse transcriptase PCR (Q-PCR) analysis, RNA-sequencing and data analysis, TUNEL assay, transmission electron microscopy, etc., were performed as described in Supporting Experimental Procedure.

### Puromycin labeling

Cells were treated with DMSO, Tg, or CHX for the indicated time before labeling. Then, the media was replaced with a labeling medium containing 10 mg/ml of puromycin for 30 min. Cells were lysed, and protein concentration was measured by bicinchoninic acid (BCA) assay. Equal amounts of protein were loaded into the gel and transferred to the membrane. Signals were detected by an anti-puromycin antibody (Sigma, MABE343). The signal from total protein loading was detected by Coomassie blue staining as a control.

### Phos-tag analysis

Cell protein lysates for Phos-tag analysis were prepared as described above and modified from our previous report [10] with the following running conditions: 15 mA for 15 min followed by 5 mA for 9.5 h for PERK using 11.5 µM Phos-tag (APExBIO Acrylamide, Houston, TX), and 100 V for 3 h for IRE1α using 75 μM Phos-tag.

### Animal study

Male C57BL/6J mice aged 8–14 weeks were purchased from Guangdong Medical Laboratory Animal Center. All mice were maintained under a standard humidity- and temperature-controlled environment on a 12-h light/dark cycle, with free access to food and water. The Institutional Animal Use and Care Committee of Sun Yat-sen University reviewed and approved the animal protocol. Mice were randomized into each group and were injected with shRBBP8 adenovirus (pADV-U6-shRBBP8-CMV-EGFP) and control adenovirus (pDKD-CMV-eFGP-U6-shRNA) by tail vein injection at 1–4 × 10^9^ PFU/mouse. The sample size of mice used in this study was estimated based on previous publications, and no blinding was done for the animal studies. For inducing ER stress in vivo, mice were injected intraperitoneally with 1 mg/kg body weight of tunicamycin for 6 or 24 h before being sacrificed. Plasma ALT and AST levels were measured by ALT Assay Kit (C009-2-1, Nanjing Jiancheng, China) and AST Assay Kit (C010-2-1, Nanjing Jiancheng, China), respectively.

### Human liver samples

The HCC liver biopsies were obtained from biopsy-proven and medical imaging-proven hepatocellular carcinoma patients. The normal control liver biopsies were obtained from patients without HCC who underwent surgery for excision of hepatic hemangioma in The Third Affiliated Hospital, Sun Yat-sen University. Exclusion criteria included known acute or chronic liver disease, except for viral hepatitis, obesity or type 2 diabetes mellitus, excessive alcohol ingestion, or pharmacological treatments. All patients were given written consent for their tissues to be collected. The study of these specimens was approved by the Ethics Committee of The Third Affiliated Hospital, Sun Yat-sen University, and was conducted in accordance with the 1975 Declaration of Helsinki.

### Statistical analysis

Results were statistically compared using the ordinary one-way ANOVA and two-way ANOVA followed by different multiples comparison post-tests (Tukey’s multiple comparison test or Bonferroni’s multiple comparison test). When pertinent, a Student’s *t*-test was performed for unpaired or paired groups. In all plots, *p* values are indicated: *p < 0.05, **p < 0.01, ***p < 0.001 were considered significant.

## Supplementary information

Author Agreements

supplementary file

Original Data File of uncrossed images

Checklist

## Data Availability

All data are available in the main text or supplementary materials or deposited in a public database. RNA-seq and CRISPR screening data sharing are granted upon request.
