# Supplementary material for: CRISPR screen identifies the role of RBBP8 in mediating unfolded protein response induced liver damage through regulating protein synthesis
Source: Cell Death Dis. 2023 Aug 18;14(8):531. doi: 10.1038/s41419-023-06046-x (PMC10435451; doi:10.1038/s41419-023-06046-x)
Supplement: Supplementary file 1 — Author Agreements [file 41419_2023_6046_MOESM1_ESM.pdf]

Heting Wang

Re: Re: Required: Agreement of the authorship for RBBP8-liver disease on Cell Death and Diseases (CDDIS-22-4643)

2023-07-30 20:16:51

发件人： "王何婷" <wanght36@mail.sysu.edu.cn>

收件人： "Yubao ZHENG" <zhybao@mail.sysu.edu.cn>

抄　送： "石国军" <shigj6@mail.sysu.edu.cn> panxy56@mail.sysu.edu.cn yang.zhang@stjude.org chjning@mail.sysu.edu.cn wenshy7@mail2.sysu.edu.cn

.. [↓还有14个联系人]

Yes, I agree with my authorship and the additions and deletions on the authorship list.

Best,

Heting

Xuya Pan

Re: Required: Agreement of the authorship for RBBP8-liver disease on Cell Death and Diseases (CDDIS-22-4643)

2023-07-30 21:30:30

发件人： "潘序雅" <panxy56@mail.sysu.edu.cn>

收件人： "石国军" <shigj6@mail.sysu.edu.cn>

Yes, I agree with my authorship and the additions and deletions on the authorship list.

> -----原始邮件-----
> **发件人:**"石国军" <shigj6@mail.sysu.edu.cn>
> **发送时间:**2023-07-26 10:18:58 (星期三)
> **收件人:** wanght36@mail.sysu.edu.cn, panxy56@mail.sysu.edu.cn, yang.zhang@stjude.org, chjning@mail.sysu.edu.cn, wenshy7@mail2.sysu.edu.cn, wangj828@mail.sysu.edu.cn, gaor25@mail2.sysu.edu.cn, yangjf26@mail2.sysu.edu.cn, zhiyp@mail2.sysu.edu.cn, wensy9@mail2.sysu.edu.cn, zhybao@mail.sysu.edu.cn, liting55@mail2.sysu.edu.cn, aiheying@mail.sysu.edu.cn, hexm26@mail.sysu.edu.cn, zhuyanh2@mail.sysu.edu.cn, luyan36@mail.sysu.edu.cn, chunliang.li@stjude.org, chyanm@mail.sysu.edu.cn, xiangxx5@mail2.sysu.edu.cn, Jin.Wang2@UTSouthwestern.edu
> **抄送:** shigj6@mail.sysu.edu.cn
> **主题:** Required: Agreement of the authorship for RBBP8-liver disease on Cell Death and Diseases (CDDIS-22-4643)

Xiaoxin Xiang

Re:Required: Agreement of the authorship for RBBP8-liver disease on Cell Death and Diseases (CDDIS-22-4643)

2023-07-26 21:32:00

发件人： "向晓昕" <xiangxx5@mail2.sysu.edu.cn>

收件人： "石国军" <shigj6@mail.sysu.edu.cn>

Yes, I agree with my authorship and the additions and deletions on the authorship list.

------------------ Original ------------------

From: "石国军"<shigj6@mail.sysu.edu.cn>;
Date: Wed, Jul 26, 2023 10:19 AM
To: "wanght36"<wanght36@mail.sysu.edu.cn>; "panxy56"<panxy56@mail.sysu.edu.cn>; "yang.zhang"<yang.zhang@stjude.org>; "chjning"<chjning@mail.sysu.edu.cn>; "wenshy7"<wenshy7@mail2.sysu.edu.cn>; "wangj828"<wangj828@mail.sysu.edu.cn>; "gaor25"<gaor25@mail2.sysu.edu.cn>; "yangjf26"<yangjf26@mail2.sysu.edu.cn>; "zhiyp"<zhiyp@mail2.sysu.edu.cn>; "wensy9"<wensy9@mail2.sysu.edu.cn>; "zhybao"<zhybao@mail.sysu.edu.cn>; "liting55"<liting55@mail2.sysu.edu.cn>; "aiheying"<aiheying@mail.sysu.edu.cn>; "hexm26"<hexm26@mail.sysu.edu.cn>; "zhuyanh2"<zhuyanh2@mail.sysu.edu.cn>; "luyan36"<luyan36@mail.sysu.edu.cn>; "chunliang.li"<chunliang.li@stjude.org>; "陈燕铭"<chyanm@mail.sysu.edu.cn>; "向晓昕"<xiangxx5@mail2.sysu.edu.cn>; "Jin.Wang2"<Jin.Wang2@UTSouthwestern.edu>;
Cc: "shigj6"<shigj6@mail.sysu.edu.cn>;
Subject: Required: Agreement of the authorship for RBBP8-liver disease on Cell Death and Diseases (CDDIS-22-4643)

Yang Zhang

Re: Required: Agreement of the authorship for RBBP8-liver disease on Cell Death and Diseases (CDDIS-22-4643)

2023-07-26 23:16:31

发件人： "Zhang, Yang" <Yang.Zhang@STJUDE.ORG>

收件人： "Li, Liang" <Chunliang.Li@STJUDE.ORG> "石国军" <shigj6@mail.sysu.edu.cn> "王何婷" <wanght36@mail.sysu.edu.cn> "panxy56@mail.sysu.edu.cn" <panxy56@mail.sysu.edu.cn> "chjning@mail.sysu.edu.cn" <chjning@mail.sysu.edu.cn> .. [↓还有15个联系人]

抄　送： "石国军" <shigj6@mail.sysu.edu.cn>

Yes, I agree with my authorship and the additions and deletions on the authorship list.

Best,

Yang

Jianning Chen

Re: Required: Agreement of the authorship for RBBP8-liver disease on Cell Death and Diseases (CDDIS-22-4643)

2023-07-30 20:23:44

发件人： "陈健宁" <chjning@mail.sysu.edu.cn>

收件人： "石国军" <shigj6@mail.sysu.edu.cn>

抄　送： "王何婷" <wanght36@mail.sysu.edu.cn> panxy56@mail.sysu.edu.cn yang.zhang@stjude.org wenshy7@mail2.sysu.edu.cn wangj828@mail.sysu.edu.cn

.. [↓还有14个联系人]

Dear Prof. Shi,

Congratulations!

Yes, I agree with my authorship and the additions and deletions on the authorship list

Yours sincerely,

Jianning Chen

Shiyi Wen

回复:Required: Agreement of the authorship for RBBP8-liver disease on Cell Death and Diseases (CDDIS-22-4643)

2023-07-30 20:51:23

发件人： "温诗怡" <wenshy7@mail2.sysu.edu.cn>

收件人： "石国军" <shigj6@mail.sysu.edu.cn>

Yes, I agree with my authorship and the additions and deletions on the authorship list.

Best,
Shiyi Wen

Jin Wang

RE: Required: Agreement of the authorship for RBBP8-liver disease on Cell Death and Diseases (CDDIS-22-4643)

2023-07-31 00:14:10

发件人： "Wang, Jin" <Jin.Wang@STJUDE.ORG>

收件人： "石国军" <shigj6@mail.sysu.edu.cn>

Yes, I agree with my authorship and the additions and deletions on the authorship list.

From: 石国军 <shigj6@mail.sysu.edu.cn>
Sent: Sunday, July 30, 2023 9:14 AM
To: Wang, Jin <Jin.Wang@STJUDE.ORG>
Subject: Required: Agreement of the authorship for RBBP8–liver disease on Cell Death and Diseases (CDDIS–22–4643)

Rong Gao

回复：Required: Agreement of the authorship for RBBP8-liver disease on Cell Death and Diseases (CDDIS-22-4643)

2023-07-27 10:56:26

发件人： "高荣" <gaor25@mail2.sysu.edu.cn>

收件人： "石国军" <shigj6@mail.sysu.edu.cn>

Yes, I agree with my authorship and the additions and deletions on the authorship list

Best,
Rong gao

Jifeng Yang

Re: Required: Agreement of the authorship for RBBP8-liver disease on Cell Death and Diseases (CDDIS-22-4643)

2023-07-26 17:51:52

发件人： "杨吉锋" <yangjf26@mail2.sysu.edu.cn>
收件人： "石国军" <shigj6@mail.sysu.edu.cn>

Yes, I agree with my authorship and the additions and deletions on the authorship list

Yaping Zhi

Re: Required: Agreement of the authorship for RBBP8-liver disease on Cell Death and Diseases (CDDIS-22-4643)

2023-07-30 12:31:38

发件人： "智亚平" <zhiyp@mail2.sysu.edu.cn>
收件人： "石国军" <shigj6@mail.sysu.edu.cn>

Yes, I agree with my authorship and the additions and deletions on the authorship list.

Yaping Zhi

Siyin Wen

Re:Required: Agreement of the authorship for RBBP8-liver disease on Cell Death and Diseases (CDDIS-22-4643)

2023-07-26 20:19:23

发件人： "文思颖" <wensy9@mail2.sysu.edu.cn>
收件人： "石国军" <shigj6@mail.sysu.edu.cn>

Yes, I agree with my authorship and the additions and deletions on the authorship list.

------------------ Original ------------------

From: "石国军"<shigj6@mail.sysu.edu.cn>;
Date: Wed, Jul 26, 2023 10:19 AM
To: "wanght36"<wanght36@mail.sysu.edu.cn>; "panxy56"<panxy56@mail.sysu.edu.cn>; "yang.zhang"<yang.zhang@stjude.org>; "chjning"<chjning@mail.sysu.edu.cn>; "wenshy7"<wenshy7@mail2.sysu.edu.cn>; "wangj828"<wangj828@mail.sysu.edu.cn>; "gaor25"<gaor25@mail2.sysu.edu.cn>; "yangjf26"<yangjf26@mail2.sysu.edu.cn>; "zhiyp"<zhiyp@mail2.sysu.edu.cn>; "文思颖"<wensy9@mail2.sysu.edu.cn>; "zhybao"<zhybao@mail.sysu.edu.cn>; "liting55"<liting55@mail2.sysu.edu.cn>; "aiheying"<aiheying@mail.sysu.edu.cn>; "hexm26"<hexm26@mail.sysu.edu.cn>; "zhuyanh2"<zhuyanh2@mail.sysu.edu.cn>; "luyan36"<luyan36@mail.sysu.edu.cn>; "chunliang.li"<chunliang.li@stjude.org>; "陈燕铭老师"<chyanm@mail.sysu.edu.cn>; "xiangxx5"<xiangxx5@mail2.sysu.edu.cn>; "Jin.Wang2"<Jin.Wang2@UTSouthwestern.edu>;
Cc: "shigj6"<shigj6@mail.sysu.edu.cn>;
Subject: Required: Agreement of the authorship for RBBP8-liver disease on Cell Death and Diseases (CDDIS-22-4643)

Yubao Zheng

Re: Required: Agreement of the authorship for RBBP8-liver disease on Cell Death and Diseases (CDDIS-22-4643)

2023-07-30 12:06:33

发件人： "Yubao ZHENG" <zhybao@mail.sysu.edu.cn>
收件人： "石国军" <shigj6@mail.sysu.edu.cn>
抄 送： "王何婷" <wanght36@mail.sysu.edu.cn> panxy56@mail.sysu.edu.cn yang.zhang@stjude.org chjning@mail.sysu.edu.cn wenshy7@mail2.sysu.edu.cn
.. [↓还有14个联系人]

Yes, I agree with my authorship and the additions and deletions on the authorship list.

Yubao Zheng

Ting Li

回复：Required: Agreement of the authorship for RBBP8-liver disease on Cell Death and Diseases (CDDIS-22-4643)

2023-07-26 10:59:54

发件人： "李婷" <liting55@mail3.sysu.edu.cn>

收件人： "石国军" <shigj6@mail.sysu.edu.cn>

Yes, I agree with my authorship and theadditions and deletions onthe authorship list.

Heying Ai

Re: Required: Agreement of the authorship for RBBP8-liver disease on Cell Death and Diseases (CDDIS-22-4643)

2023-07-30 22:49:40

发件人： "艾鹤英" <aiheying@mail.sysu.edu.cn>

收件人： "石国军" <shigj6@mail.sysu.edu.cn>

抄　送： "王何婷" <wanght36@mail.sysu.edu.cn> panxy56@mail.sysu.edu.cn yang.zhang@stjude.org chjning@mail.sysu.edu.cn wenshy7@mail2.sysu.edu.cn

.. [↓还有14个联系人]

Yes, I agree with my authorship and the additions and deletions on the authorship list.

Best,

Heying Ai

-----原始邮件-----
发件人:"石国军" <shigj6@mail.sysu.edu.cn>

Xuemin He

Re: Required: Agreement of the authorship for RBBP8-liver disease on Cell Death and Diseases (CDDIS-22-4643)

2023-07-26 10:21:59

发件人： "何学敏" <hexm26@mail.sysu.edu.cn>

收件人： "石国军" <shigj6@mail.sysu.edu.cn>

Congratulations!

Yes, I agree with my authorship and the additions and deletions on the authorship list

Xuemin

Yanhua Zhu

Re: Re: Required: Agreement of the authorship for RBBP8-liver disease on Cell Death and Diseases (CDDIS-22-4643)

2023-07-27 10:49:46

发件人： "朱延华" <zhuyanh2@mail.sysu.edu.cn>

收件人： "Zhang, Yang" <Yang.Zhang@STJUDE.ORG>

抄　送： "Li, Liang" <Chunliang.Li@STJUDE.ORG> "石国军" <shigj6@mail.sysu.edu.cn> "王何婷" <wanght36@mail.sysu.edu.cn> "panxy56@mail.sysu.edu.cn" <panxy56@mail.sysu.edu.cn> "chjning@mail.sysu.edu.cn" <chjning@mail.sysu.edu.cn> .. [↓还有14个联系人]

Yes, I agree with my authorship and the additions and deletions on the authorship list. Congratulations to everyone involved in this wonderful project.

Best,

Yanhua Zhu

Yan Lu

回复：Required: Agreement of the authorship for RBBP8-liver disease on Cell Death and Diseases (CDDIS-22-4643)

2023-07-27 07:28:42

发件人： "luyan36@mail.sysu.edu.cn" <luyan36@mail.sysu.edu.cn>

收件人： "石国军" <shigj6@mail.sysu.edu.cn> "王何婷" <wanght36@mail.sysu.edu.cn> "panxy56@mail.sysu.edu.cn" <panxy56@mail.sysu.edu.cn> "yang.zhang@stjude.org" <yang.zhang@stjude.org> "chjning@mail.sysu.edu.cn" <chjning@mail.sysu.edu.cn> .. [↓还有15个联系人]

Yes, I agree with my authorship and the additions and deletions on the authorship list.

Best,

Yan

Chunliang Li

Re: Required: Agreement of the authorship for RBBP8-liver disease on Cell Death and Diseases (CDDIS-22-4643)

2023-07-26 21:01:26

发件人： "Li, Liang" <Chunliang.Li@STJUDE.ORG>

收件人： "石国军" <shigj6@mail.sysu.edu.cn> "王何婷" <wanght36@mail.sysu.edu.cn> "panxy56@mail.sysu.edu.cn" <panxy56@mail.sysu.edu.cn> "Zhang, Yang" <Yang.Zhang@STJUDE.ORG> "chjning@mail.sysu.edu.cn" <chjning@mail.sysu.edu.cn> .. [↓还有15个联系人]

抄 送： "石国军" <shigj6@mail.sysu.edu.cn>

Yes, I agree with my authorship and the additions and deletions on the authorship list. Congratulations to everyone involved in this wonderful project. Cheers. Liang

Get Outlook for iOS

From: 石国军 <shigj6@mail.sysu.edu.cn>
Sent: Tuesday, July 25, 2023 9:18:58 PM
To: wanght36@mail.sysu.edu.cn <wanght36@mail.sysu.edu.cn>; panxy56@mail.sysu.edu.cn <panxy56@mail.sysu.edu.cn>; Zhang, Yang <Yang.Zhang@STJUDE.ORG>; chjning@mail.sysu.edu.cn <chjning@mail.sysu.edu.cn>; wenshy7@mail2.sysu.edu.cn <wenshy7@mail2.sysu.edu.cn>; wangj828@mail.sysu.edu.cn <wangj828@mail.sysu.edu.cn>; gaor25@mail2.sysu.edu.cn <gaor25@mail2.sysu.edu.cn>; yangjf26@mail2.sysu.edu.cn <yangjf26@mail2.sysu.edu.cn>; zhiyp@mail2.sysu.edu.cn <zhiyp@mail2.sysu.edu.cn>; wensy9@mail2.sysu.edu.cn <wensy9@mail2.sysu.edu.cn>; zhybao@mail.sysu.edu.cn <zhybao@mail.sysu.edu.cn>; liting55@mail2.sysu.edu.cn <liting55@mail2.sysu.edu.cn>; aiheying@mail.sysu.edu.cn <aiheying@mail.sysu.edu.cn>; hexm26@mail.sysu.edu.cn <hexm26@mail.sysu.edu.cn>; zhuyanh2@mail.sysu.edu.cn <zhuyanh2@mail.sysu.edu.cn>; [illegible]

Yanming Chen

Re: 回复：Re: Required: Agreement of the authorship for RBBP8-liver disease on Cell Death and Diseases (CDDIS-22-4643)

2023-07-31 22:07:59

发件人： "陈燕铭" <chyanm@mail.sysu.edu.cn>

收件人： "石国军" <shigj6@mail.sysu.edu.cn>

Yes, I agree with my authorship and the additions and deletions on the authorship list

yanming chen

Guojun Shi

Re: Required: Agreement of the authorship for RBBP8-liver disease on Cell Death and Diseases (CDDIS-22-4643)

2023-08-03 03:34:42

发件人： "石国军" <shigj6@mail.sysu.edu.cn>

收件人： "石国军" <shigj6@mail.sysu.edu.cn>

Yes, I agree with my authorship and the additions and deletions on the authorship list.

Thanks,

Guojun Shi
