# Supplementary material for: CRISPR screen identifies the role of RBBP8 in mediating unfolded protein response induced liver damage through regulating protein synthesis
Source: Cell Death Dis. 2023 Aug 18;14(8):531. doi: 10.1038/s41419-023-06046-x (PMC10435451; doi:10.1038/s41419-023-06046-x)
Supplement: Supplementary file 2 — supplementary file [file 41419_2023_6046_MOESM2_ESM.docx]

**Supporting Information for**

CRISPR screen identifies the role of RBBP8 in mediating unfolded protein response induced liver damage through regulating protein synthesis

Heting Wang^1,8^, Xuya Pan^1,8^, Xiaoxin Xiang^1,8^, Yang Zhang^2,3^, Jianning Chen^4^, Shiyi Wen^1^, Jin Wang^1^, Rong Gao^1^, Jifeng Yang^1^, Yaping Zhi^1^, Siying Wen^1^, Yubao Zheng^5^, Ting Li^1^, Heying Ai^1,^, Xuemin He^1^, Yan Lu^6^, Yanhua Zhu^1^, Chunliang Li^2,3,*^, Yanming Chen^1,*^, Guojun Shi^1,7,*^

Guojun Shi, [shigj6@mail.sysu.edu.cn](mailto:shigj6@mail.sysu.edu.cn)

Yanming Chen, [chyanm@mail.sysu.edu.cn](mailto:chyanm@mail.sysu.edu.cn)

Chunliang Li, [chunliang.li@stjude.org](mailto:chunliang.li@stjude.org)

**This PDF file includes:**

Figures S1 to S9

Supporting methods

SI References 1-17

Supplementary Figures


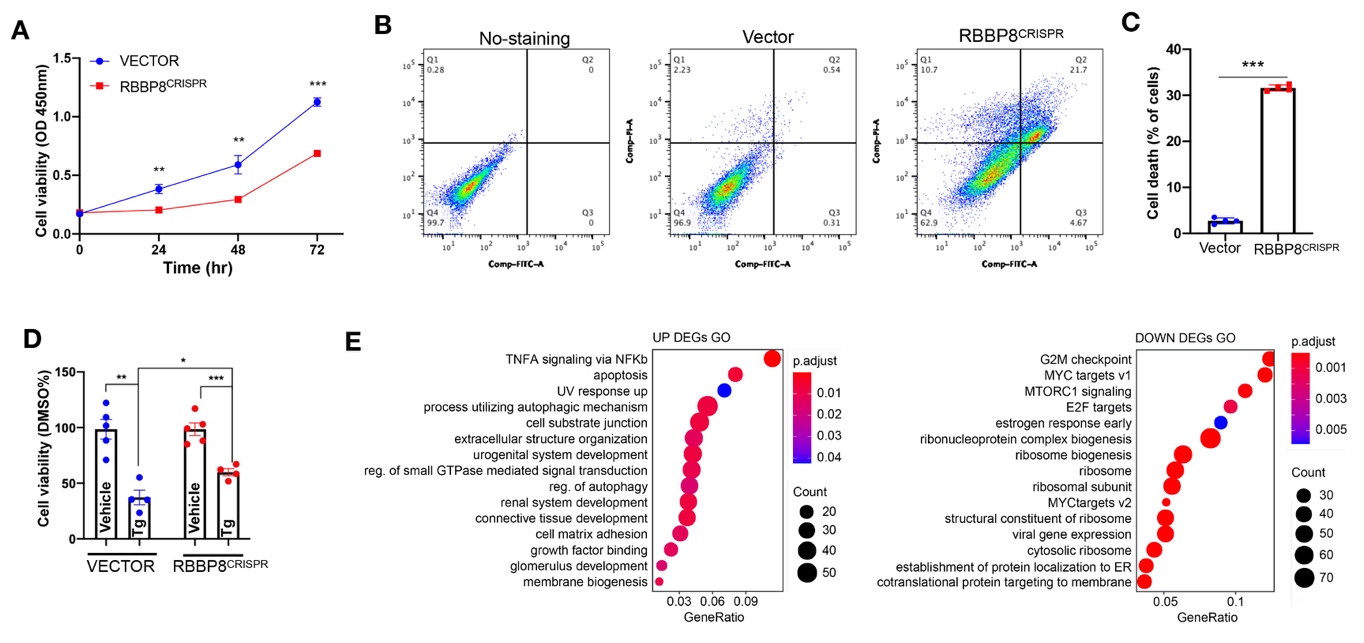


**Figure S1. RBBP8 deficiency leads to growth retardation and cell death.**

**A.** HEK293T cells were transfected with lentivirus carrying either CRISPR sgRNA against RBBP8 or empty vector, and cell proliferation was measured by CCK-8 assay.

**B-C. B,** 48 hours post lentivirus infection, HEK293T cells were stained with AnnexinV and PI, followed by flow cytometry analysis, and quantification of PI^+^ populations was shown (C).

**D.** HEK293T cells 48hr post lentivirus infection were treated with 1uM Tg for 24hr, and cell viability was measured by CCK-8 assay, and analyzed by normalization to the control of respective genotype.

Data presented as mean ± SEM,**p*< 0.05, ***p*< 0.01, ****p*< 0.001, by student’s t-test.

**E.** Gene Ontology (GO) enrichment analysis of DEGs that significantly changed under basal condition RBBP8-KO versus Control cells.


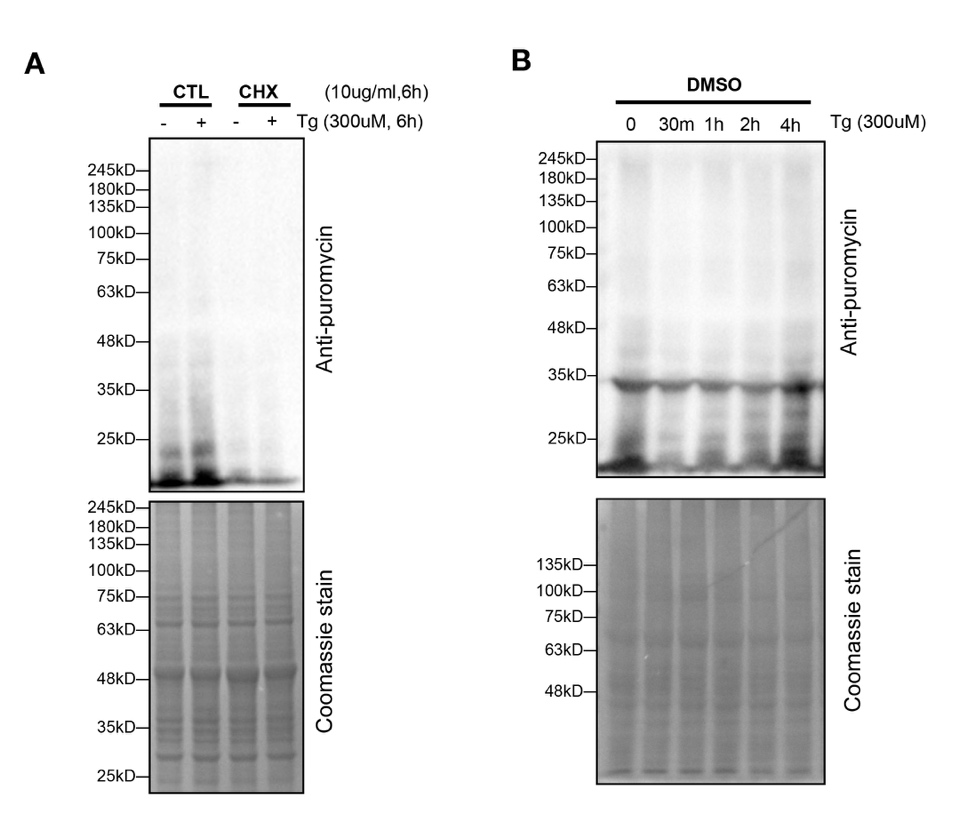


**Figure S2. RBBP8 deficiency led to reduced protein synthesis.**

**A-B.** HEK293T cells were pulse-labeled with puromycin after cycloheximide and/or Tg treatment (A) or Tg treatment along (B) for the indicated time. Immunoblotting analysis against puromycin is shown (upper) and Coomassie stain (lower) represents protein loading control.


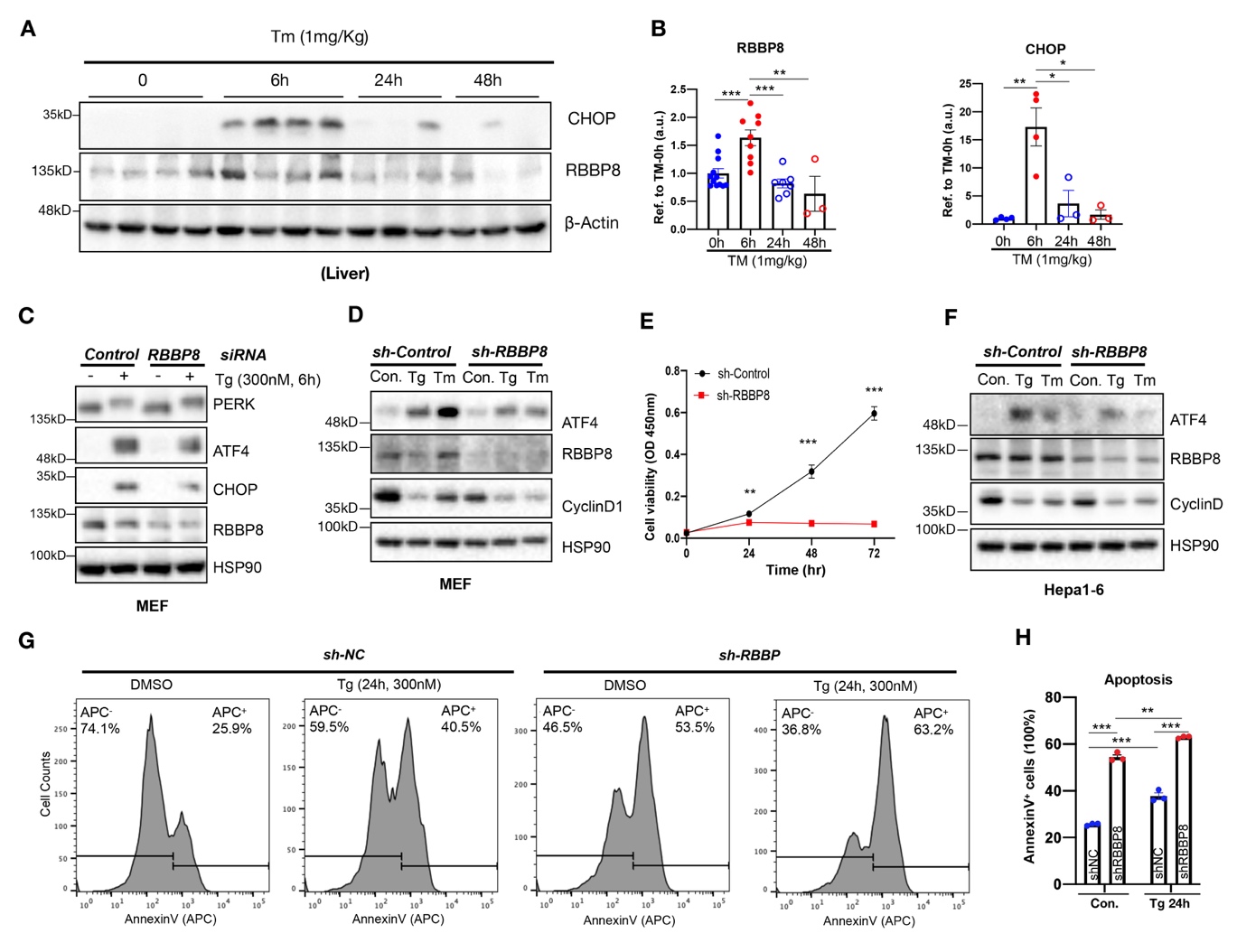


**Figure S3. Inhibition of RBBP8 by siRNA and Ad-shRNA attenuated ATF4 activation in mouse cell line.**

**A-B.** Immunoblotting analysis of RBBP8 and CHOP expression in livers from mice (8-weeks, male) followed by Tunicamycin injection (1mg/kg, i.p.) at indicated time, with quantification of RBBP8 and CHOP protein level shown in (**B**).

**C-D.** Immunoblotting analysis of RBBP8-deficient MEF cells by siRNA or Adenovirus mediated shRNA under Tg (300nM) or Tm (2.5ug/ml) treatment for 6h.

**E.** MEF cells were transfected with adenovirus-shRNA, and cell growth was measured by CCK-8 assay.

**F.** Immunoblotting analysis of RBBP8-deficient Hepa1-6 cells by adenovirus-shRNA under Tg treatment(300nM) and Tm treatment (2.5ug/ml) for 6h.

**G-H.** Hepa1-6 cells were treated with Adeno-shRBBP8 and stained with AnnexinV 48h post infection, followed by Tg treatment and flow cytometry analysis as indicated, and quantification was shown in (H).

All data represented at least 3 independent experiments. Data presented as mean ± SEM, **p*< 0.05, ***p*< 0.01 and ****p*< 0.001 by student’s t-test.


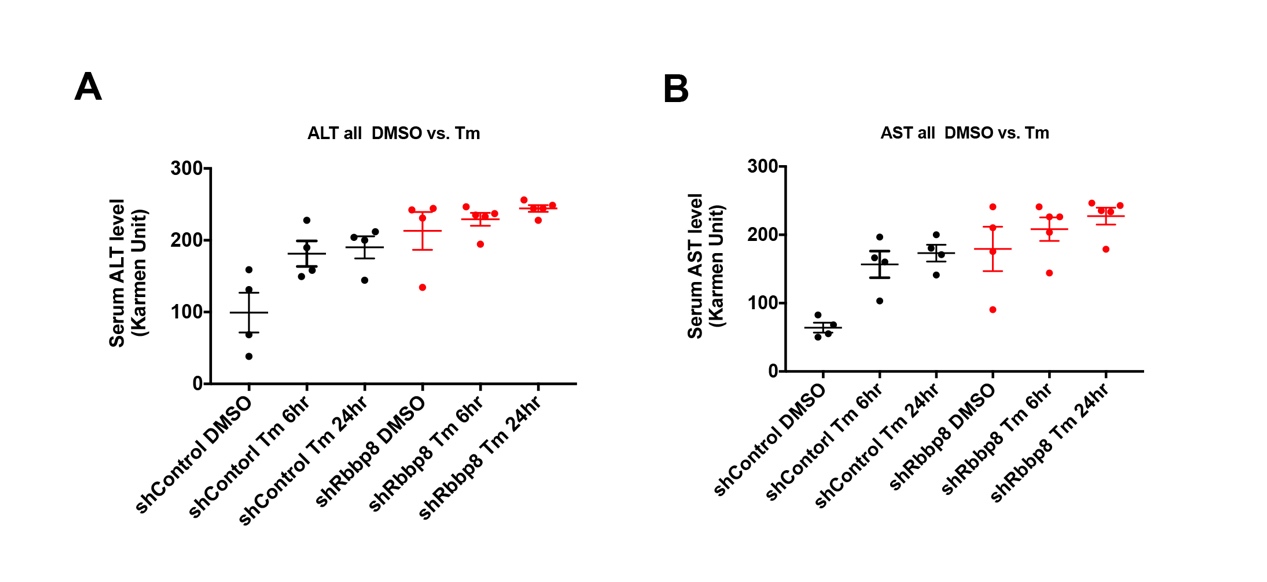


**Figure S4. Tm induced liver damage in RBBP8 deficient mice.**

**A-B.** Serum ALT levels (A) and AST (B) levels of Ad-shNC and Ad-shRBBP8 mice 16 days post virus injection. Data presented as mean ± SEM, **p*< 0.05, ***p*< 0.01***p< 0.001. n=4~5 each group.


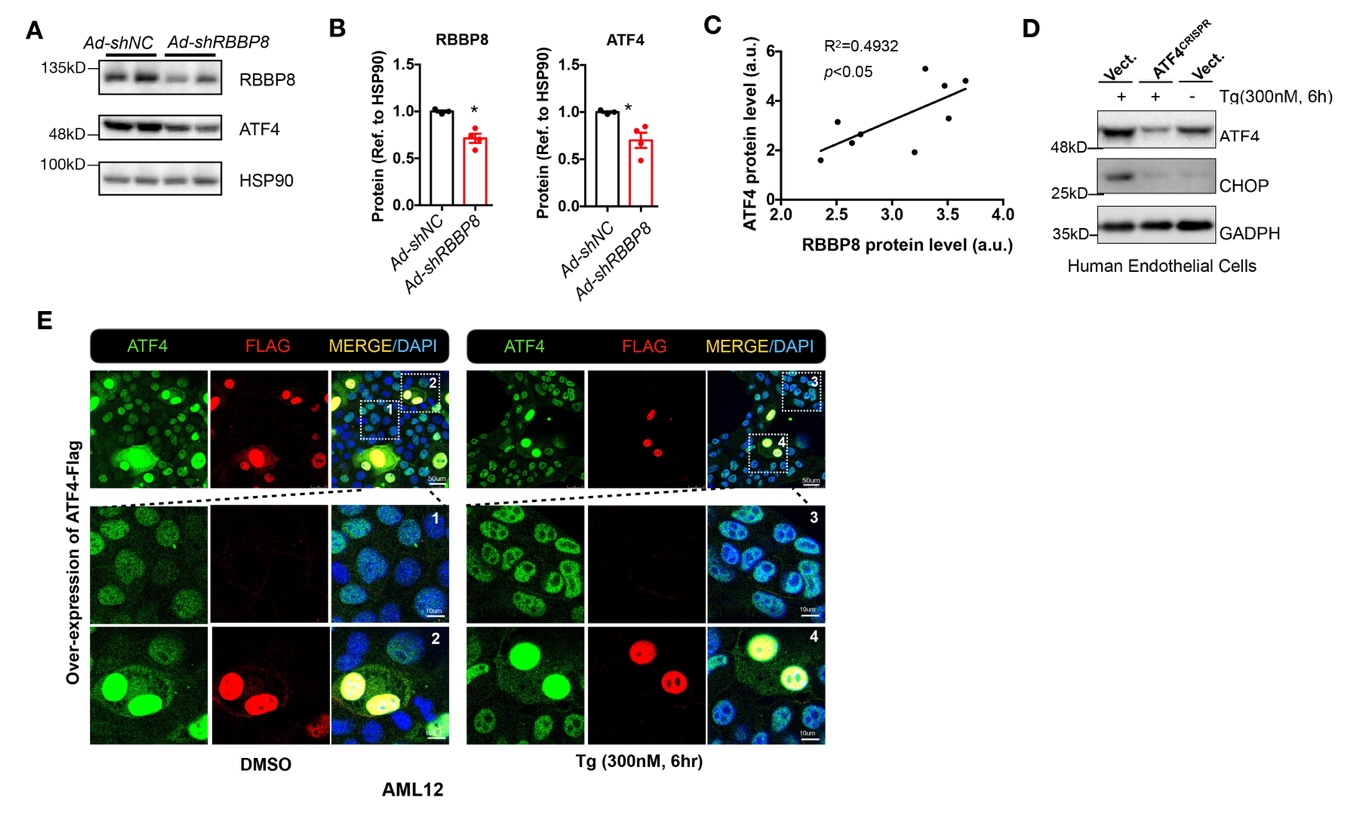


**Figure S5. RBBP8 deficiency led to reduced ATF4 expression.**

**A-B.** Immunoblotting analysis of RBBP8 and ATF4 in liver lysates of mice 16 days post adenovirus injection, and quantification was shown in B. (14-15 weeks old mice, n = 3-4 per group).

**C.** Linear regression analysis of RBBP8 and ATF4 protein level from A showed positive correlation between RBBP8 and ATF4 in livers combined with either genotype.

**D.** Immunoblotting analysis confirming the antibody specificity of ATF4 in LentiV2-ATF4^CRISPR^ plasmid transfected human endothelial cells mediated by plasmid.

**E.** Representative immunofluorescent images of ATF4 and Flag staining in AML12 cells transfected with ATF4-Flag constructs.

Data presented as mean ± SEM, **p*< 0.05, ***p*< 0.01 and ****p*< 0.001 by student’s t-test.


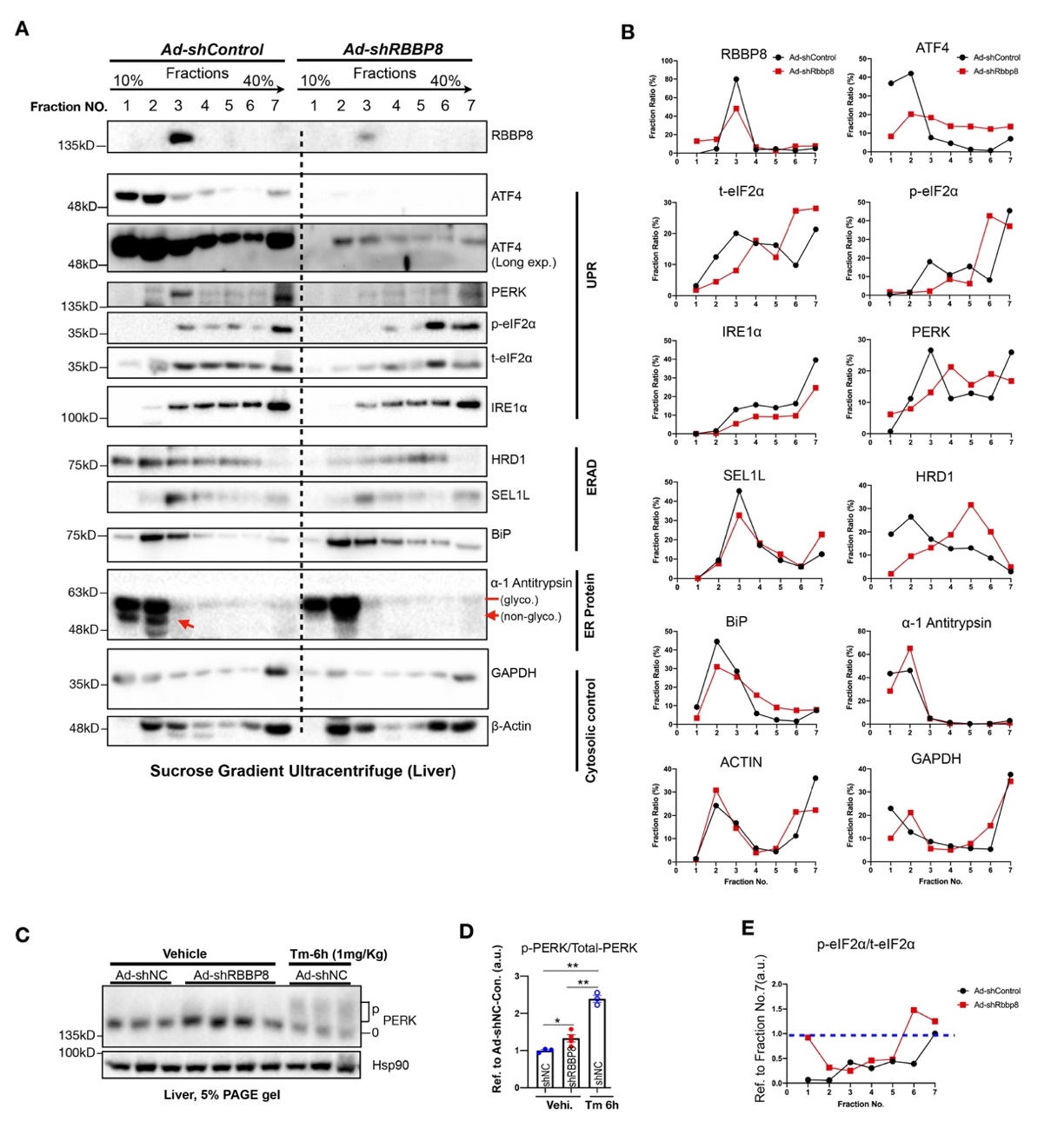


**Figure S6. ER stress related protein distribution in liver with RBBP8 deficiency analyzed by sucrose gradient ultracentrifuge.**

**A-B.** Sucrose gradient fractionation of mice liver lysates followed by Immunoblotting analysis. Fractions were collected from top (no. 1) to bottom (no. 6) and the redissolved pellet as fraction no. 7, and quantifications of respective protein distribution were shown B.

**C-D.** Immunoblotting analysis of PERK expression in livers of 14-15 weeks old mice 17 days post virus injection, followed by Tunicamycin injection (1mg/kg, i.p.). Protein expression of phosphorylated PERK and un-phosphorylated PERK was performed by 5% PAGE gel, with quantification of phosphorylated PERK protein level shown in D.

**E.** The Ratio of p-eIF2α/t-eIF2α in RBBP8 deficient and control liver lysate quantified from A and B in reference to the 7th fraction of control mice.

All data represent at least 3 independent experiments. Data presented as mean ± SEM, **p*< 0.05, ***p*< 0.01 and ****p*<0.001 by student’s t-test.


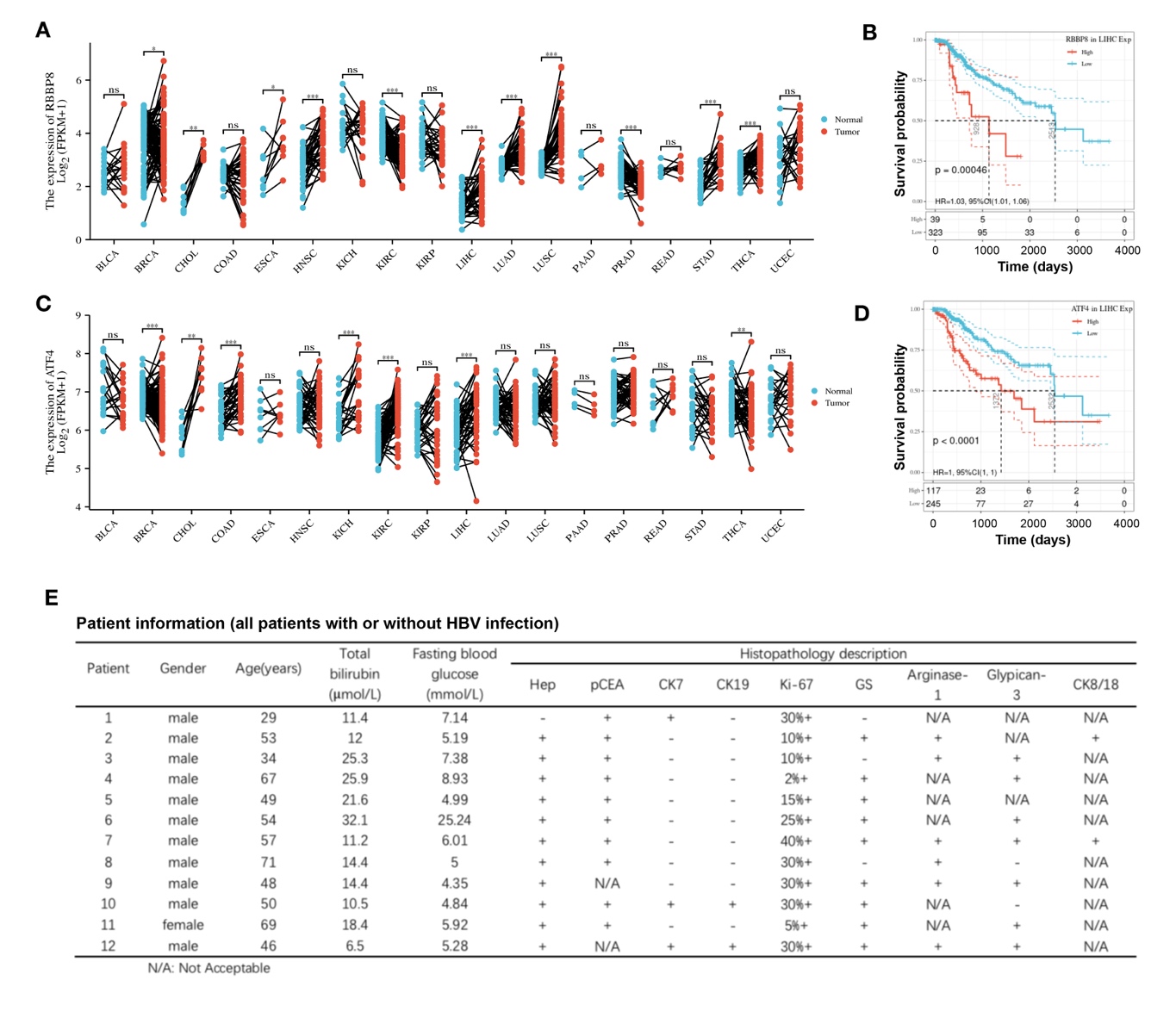


**Figure S7. Increased expression of RBBP8 and ATF4 in liver cancer and clinical information of patients analyzed in this study.**

**A-C.** Oncomine TCGA database analysis show RBBP8 (A) and ATF4(C) copy number and mRNA expression levels in various cancer tissues, respectively. n.s. for no significance, **p*< 0.05, ***p*< 0.01, and ****p*< 0.001 by student’s t-test.

**B-D.** Kaplan–Meier survival analysis of 362 liver hepatocyte cancer patients stratified by RBBP8 (B) and ATF4(D) expression level. *p*= 0.004 by log-rank (Mantel–Cox) test.


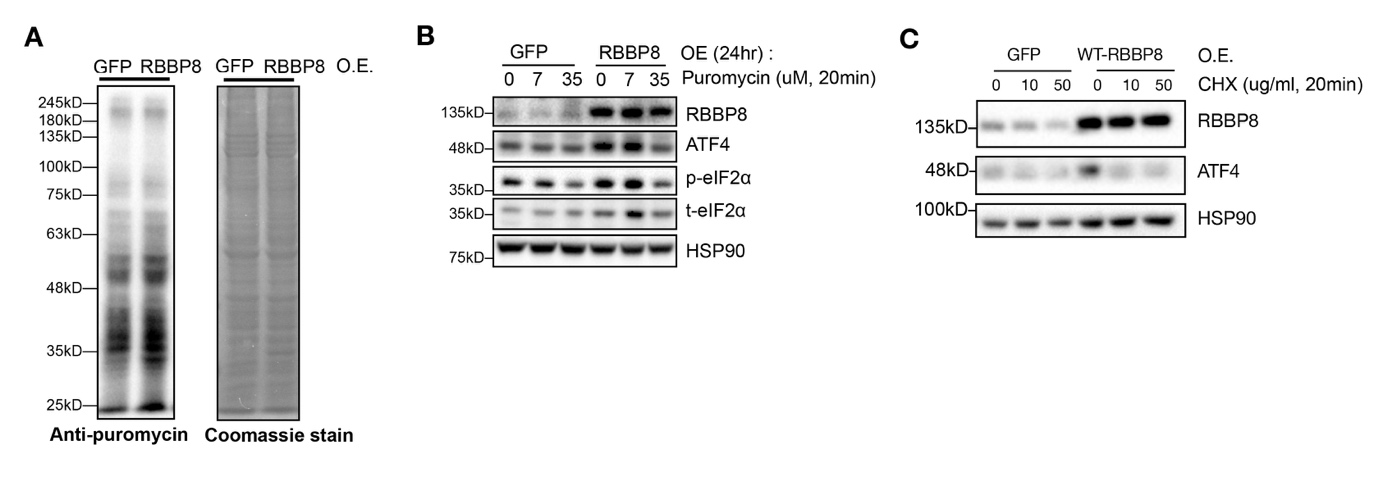


**Figure S8. RBBP8 regulates ATF4 activation through regulating protein synthesis.**

**A.** HEK293T cells were labeled with puromycin after GFP or RBBP8 over-expression. A representative blot is shown, and Coomassie stain was used as loading control.

**B-C.** Protein synthesis were inhibited by puromycin (B) or cycloheximide (CHX) (C) in HEK293T cells with GFP/RBBP8 over-expression, followed by Immunoblotting analysis.

All data represent at least 3 independent experiments.


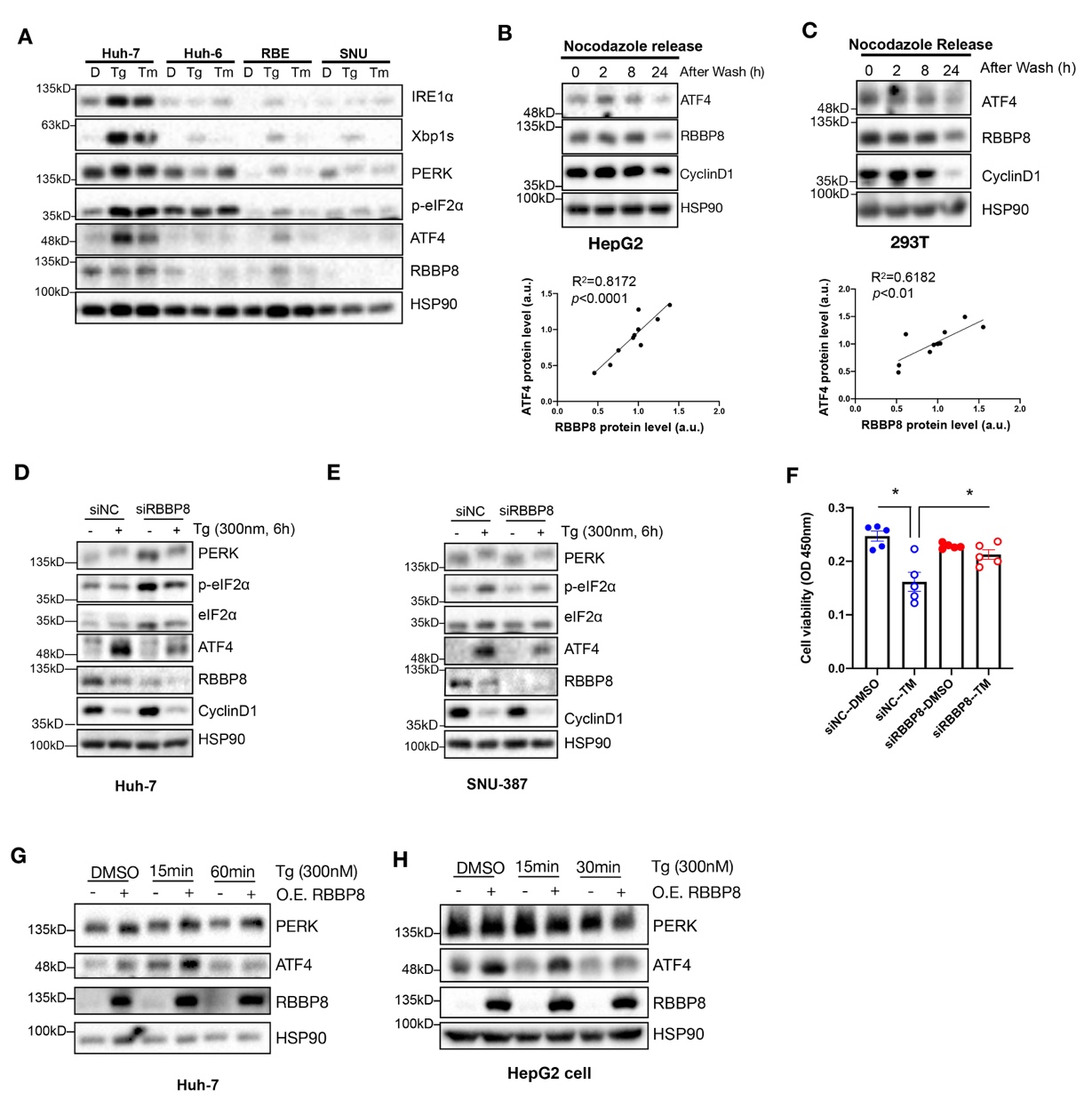


**Figure S9. Expression and function of RBBP8 in liver cancer cell lines.**

**A**. Immunoblotting analysis of UPR markers and RBBP8 expression in Huh-7, Huh-6, RBE and SNU-387cells under Tg (300nM) or Tm (2.5ug/ml) treatment for 6h. Data represent at least 2 independent experiments.

**B-C.** HepG2 (B) and HEK293T (C) cells were treated with nocodazole for 12 h and released for the indicated time, followed by immunoblotting analysis. Quantification of ATF4 and RBBP8 protein levels were analyzed for correlation analysis shown below by liner regression. Data represents at least 3 independents.

**D-E.** Immunoblotting analysis of RBBP8-deficient Huh-7 cells (D) and SNU-387 cells (E) by siRNA under Tg treatment. Data represents at least 3 independent experiments.

**F.** Cell viability of Huh-7 cells 48h post siRNA transfection, and then treated with 2.5ug/ml Tm for 24hr, followed by CCK-8 assay. Data presented as mean ± SEM, n = 4-5, **p*< 0.05.

**G-H.** Immunoblotting analysis for ATF4 activation in Hun-7 (G) and HepG2 (H) cells transfected with RBBP8 plasmid under Tg treatment for indicated time**.** Data represents at least 2 independent experiments.

**Methods and material**

**Cell culture and treatment**. SEM cells (ACC-546, DSMZ), SNU-387, and RBE cells were maintained in a standard RPMI-1640 medium (Lonza) containing 10% fetal bovine serum (HyClone), and 1% penicillin/streptomycin (Thermo Fisher Scientific) at 37°C, 5% CO2 atmosphere and 95% humidity. All passages of cells used in this study were mycoplasma-free and cell identity was confirmed by STR analysis before being used for experiments. MCF7, HEK293T, HepG2, Hepa1-6, MEF, Huh-7, Huh-6, and LX-2 cells were cultured as described above except using DMEM (Corning).

**CRISPR screening.** The genome-scale human CRISPR KO H3 library (Addgene #133914) was developed by Myles Brown and Shirley Liu labs and purchased from Addgene. This library is in an all-in-one vector modified from lentiCRISPRv2 format (a vector that contains both Cas9 and sgRNA sequences), containing sgRNAs targets more than 18, 000 genes with 6 sgRNAs per gene along with control sgRNAs. The reporter plasmids pLVX-ATF4 mScarlet NLS (Addgene #115969) was purchased from Addgene. The reporter constructed were packaged into lentivirus according to our previously published protocol (12, 20). SEM cells were infected with the lentiviral particles and selected for puromycin to establish the stably integrated reporter cell lines. Expression of reporter fluorescence was confirmed by flow cytometry analysis. Suspension-cultured SEMs were collected by centrifugation at 800X*g*, filtered through a 70-µm filter, and analyzed for mScarlet on a BD FACS Aria III flow cytometer with a negative control. The 4,6-diamidino-2-phenylindole (DAPI) staining was conducted prior to sorting to exclude dead cells. High-titer lentiviral H3 library stocks were generated in 293T cells as previously described. The ATF4 reporter cell lines were infected by the pooled H3 sgRNA library at low M.O.I (~0.3). About 3X10^8^ infected cells (~30% infection efficiency) were sorted for mScarlet^High^ and mScarlet^Low^ populations at day 7 post infection. The sgRNA sequences were recovered by genomic PCR analysis and deep sequencing using HiSeq for single-end 150-bp read length (Illumina) with about 10 million reads for each sample. The raw FASTQ data were de-barcoded and mapped to the original reference H3 sgRNA library. The differentially enriched sgRNAs were defined by comparing normalized counts between sorted cells in the top 10% and those in the bottom 10% of mScarlet or mNeonGreen-expressing bulk populations. Two independent replicate screens were performed with each reporter cell line. Normalized counts for each sgRNA were extracted and used to identify differentially enriched sgRNA by DESeq2 algorithm (37). The combined analysis of seven sgRNAs against each human transcription factor was conducted by using the MAGeCK algorithm (22).

**Western blot and antibodies.** Protein extraction from cell and tissue lysates and Western blotting following SDS–PAGE separation were performed as previously described (38). The signal intensity of the target protein bands was quantitatively analyzed using the Image Lab software (Bio-Rad, Hercules, CA, USA), and protein levels among samples were normalized to Hsp90 or β‐actin as loading controls. The following antibodies were used for Western blotting and immunostaining: HSP90 (rabbit, #4877S; Cell Signaling Technology.), RBBP8 (rabbit, ab70163, Abcam), RBBP8 (mouse, 61141,Active Motif), ATF4 (rabbit, #11815, Cell Signaling Technology; sc-390063, Santa Cruz Biotechnology Inc.); p-eIF2α (rabbit, #3398S, Cell Signaling Technology); EIf2α (rabbit, #5324S, Cell Signaling Technology); CHOP (mouse, #2895S, Cell Signaling Technology); PERK (rabbit, #3192S, Cell Signaling Technology); CyclinD1(rabbit, A19038, Abclonal); Xbp1s(rabbit, #83418S, Cell Signaling Technology); β-actin (mouse, A5441, Sigma); IRE1α (rabbit, #2118s, Cell Signaling Technology); GADD34 (rabbit, 10449-1-AP, Proteintech), Puromycin (mouse, MABE343, Merck millipore), HRD1(rabbit, 13473-1-AP, Proteintech), SEL1L(rabbit, ab78298, Abcam), Bip (rabbit, ab21685, Abcam), α-1-antiTrypsin(rabbit, 16382-1-AP, Proteintech), p-S6K(rabbit, 28988-1-AP, Proteintech), t-S6K (rabbit, #9202, Cell Signaling Technology),p-4EBP1(rabbit, #2855, Cell Signaling Technology), t-4EBP1(mouse, 60246-1-lg, Proteintech); Ki67 (ab156956, Abcam; GB121141, Servicebio Inc.). The secondary antibodies used for Western blotting were: goat anti-rabbit IgG HRP and goat anti-mouse IgG HRP (both from Bio-Rad).

**SiRNA, shRNA and overexpression of target genes.** Double-stranded small interfering RNA (siRNA) oligonucleotides against human or mouse RBBP8 were synthesized by Obio Technology (Guangzhou, China) and a non-silencing siRNA was used as the negative control. The sequence for targeting the human RBBP8 gene was GCUAAAACAGGAACGAAUCdTdT. Transfection was carried out using Lipofectamine 3000 (Invitrogen, Carlsbad, CA, USA) and culture in Opti-MEM for 24 h. Cells were recovered after being left in a normal culture medium 72h for western blot or 24h for QPCR then treated with Tg or DMSO. Human WT RBBP8 inserted on pcDNA3.1 plasmid was provided by HedgehogBio Science and Technology Ltd. Human E157K, K467A RBBP8 mutants were generated based on WT-RBBP8 pcDNA3.1 plasmid by using PCR. The shRNA-adenovirus (pADV-U6-shRBBP8-CMV-EGFP and pDKD-CMV-eFGP-U6-shRNA) for the animal study were constructed and amplified by OBIO Technology Ltd, and the sequence for shRBBP8 was 5’-GCAACCAAGAUACGUCCUU-3’.

**Histological analysis and Immunofluorescence staining.** For immunostaining of MCF7 cells, a 12-mm-diameter cover glass was coated with 0.1% poly-L-lysine solution (P8920; Sigma-Aldrich). Cells (1×10^4^) were placed on a coated cover glass in a 6-well plate and treated with thapsigargin (300nM) or DMSO for 6 hours. Cells were then fixed with 4% formaldehyde for 15 minutes at room temperature and then washed with PBS and permeabilized using 0.5% Triton X-100 for 15 minutes at room temperature, followed by incubation in blocking solution. Cells were stained as described above. For Oil Red O and Nile Red staining, mouse liver tissue was frozen at optimal cutting temperature (OCT), sectioned (5 μm thick), and stained with Oil Red O (1320-06, Sigma-Aldrich) or Nile Red (7385-67-3, Sigma-Aldrich) to evaluate hepatic lipid content. Liver tissues were fixed in 4% formaldehyde, and processed paraffin embedding, sectioning, and H&E staining. For immunofluorescence staining, paraffin-embedded liver sections were dewaxed, rehydrated, boiled in 1mM Tris-EDTA for antigen retrieval, and then incubated in a blocking solution (3% goat serum and 0.5% Triton X-100 in 0.05M phosphate-buffered saline [PBS]) for 20 minutes at room temperature. After blocking, liver sections were incubated overnight with primary antibodies at 4 ℃ and, following 3 washes with PBST (0.2% Triton X-100 in 0.05M PBS), were incubated with secondary antibodies for 2 hours at room temperature. Counterstaining and mounting were performed with a mounting medium containing DAPI (Vector H-1400) and a Fisherfinest Premium Coverslip (12-548-5p; Thermo Fisher Scientific).

**Immunoreactivity signal quantitation.** Fluorescent samples were imaged with a Leica SP8 confocal microscope, and imaging parameters were identical within each set of samples. Liver H&E sides and TUNEL assay slides were scanned using an automatic digital slide scanner (Pannoramic MIDI). To quantify immunostaining signal using FIJI, individual cells were outlined, and fluorophore intensity was measured as an average gray value (intensity/area) in arbitrary units.

**Cell cycle and Cell viability analysis.** Flow cytometry analysis of the cell cycle. 293T cells at the logarithmic growth phase were seeded in 6-well plates. After reaching 50% confluence, the adherent cells were cultured in lentivirus contained serum-free medium for 24 h and then were cultured in DMEM supplemented with 10% FBS. After 48 h, the cells were drug treated as indicated time then digested and harvested with 250 μl trypsin. The cell pellet was obtained following centrifugation for 3 min at 4 ̊C and 978 x g, and was re-suspended with 500 μl ice-cold PBS on ice, followed by resuspension in 70% ethanol at 4 ̊C for 30 min. Finally, 1 ml propidium iodide (PI) staining solution (Beyotime, C1052, China) was added to the samples, and then the associated data were analyzed on a FACScan (Becton-Dickinson, San Francisco, CA, USA). Results were acquired from 10,000 cells. PI unstained cells were used as a negative control. **2**93T and Hepa1-6 cells (5 × 10^3^ per well) were seeded in 96- well plates overnight and then further incubated for 1, 2, 3, and 4 days, respectively. For the CCK-8 assay(39), cells were added with 10 ml Cell Counting Kit-8 (CCK-8; Dojindo, Kumamoto, Japan) solution and furtherly incubated at 37°C for 2 h. The absorbance of each well was measured at 450 nm by using a Multiskan Go spectrophotometer (Thermo Fisher Scientific, Inc.). The drug-mediated cell proliferation arrest with the indicated doses was calculated by comparing the absorbance versus that of no treatment control.

**Cell synchronization analysis.** Cells were pretreated with nocodazole (200 ng/ml; Aldrich-Sigma) for 12 h to arrest cells at the G2/M-phase and changed to a fresh medium to indicate time to synchronize cells to different cell phase(33). Cells were lysed and protein concentration was measured by bicinchoninic acid (BCA) assay.

**Annexin V staining assay.** 293T or Hepa1-6 cell lines at 1 × 10^5^ cells/ml were cultured in 6-well plates in the presence of Tg for 24 h. The quantification of the apoptotic cells was measured by Annexin V-FITC or Annexin V-APC (fluorescein isothiocyanate)/PI (propidium iodide) co-staining assay. Briefly, at the end of the 24 h incubation, the cells were harvested and centrifuged at 1500 rpm for 5 min. The pellet was resuspended in a 100 μl binding buffer containing 3 μl Annexin V-FITC and then incubated at room temperature for 30 min in the dark. 3ul PI in 200 μl binding buffer was added to each of the tubes and incubated for 5 min. Finally, the cells were analyzed by flow cytometry (Becton-Dickinson, San Francisco, CA, USA). Results were acquired from 10,000 cells. Both Annexin V-FITC and PI unstained cells were used as a negative control.

**Edu staining assay.** EdU cell proliferation staining was performed as following described(40) using an EdU kit (BeyoClick™ EdU Cell Proliferation Kit with Alexa Fluor 488, C0071S, Beyotime, China). Briefly, siRNA transfected 293T cells (1× 104 cells/well) were cultured on round coverslips in 12-well plates for 24 h. Subsequently, cells were incubated with EdU for 2 h, fixed with 4% paraformaldehyde for 15 min, and permeated with 0.3% Triton X-100 for another 15 min. The cells were incubated with the Click Reaction Mixture for 30 min at room temperature in a dark place and then incubated with DAPI for 10 min to counterstain the nucleus.

**Quantitative PCR and quantification.** Total RNA was extracted from 293T cells or mice liver samples using the TRIZOL reagent (Invitrogen, Carlsbad, CA, USA). Quantitative real-time (RT)-PCR was conducted using the FastKing RT kit and SuperReal PreMix Plus kit (SYBR Green) (KR116-02 and FP205-02; Tiangen Biotech Co., Ltd., Beijing, China), according to the manufacturer's instructions. The b-actin mRNA level was used as the internal control. The PCR program included one cycle at 95°C for 15 min; followed by 40 cycles at 95°C for 10 s; 60°C for 20 s; and 72°C for 30 s. Analysis of each sample was repeated three times. The primer sequences used are listed in [Supplementary Table 1](https://www.ncbi.nlm.nih.gov/pmc/articles/PMC2963107/#SD1).

**RT-PCR for Xbp1 splicing.** PCR primers were designed to encompass the splicing sequences of human Xbp1 ([Supplementary Table 1](https://www.ncbi.nlm.nih.gov/pmc/articles/PMC2963107/#SD1)). PCR products, amplified with annealing temperature at 58° C for 30 cycles, were separated by electrophoresis on a 2.5% agarose gel (Invitrogen). Quantitation of percent of splicing, defined as the ratio of Xbp1s level to total Xbp1 (Xbp1u + Xbp1s) levels, was quantitatively analyzed using the Image Lab software (Bio-Rad, Hercules, CA, USA)(41).

**RNA-Sequencing.** WT or RBBP8 KO 293T cells were treated with DMSO or 300nm of Tg for 6 hours and isolated RNA by TRIZOL buffer (Invitrogen) and stored in a refrigerator at −80°C for subsequent total RNA extraction. Furthermore, 3 μg RNA per sample was used as input for the RNA sample preparations. The RNA sequencing was performed by Novogene Inc. (Beijing, China).

**RNA-seq and data analysis** Following cell preparation, RNA was extracted and library prepared using TruSeq RNA Sample Preparation Kit (Illumina). Libraries were evaluated using an Agilent DNA1000 kit (Agilent Technologies) and quantified by Invitrogen Qubit HS cDNA Kit (Invitrogen). Libraries were sequenced as pair-end 150 bp on an Illumina HiSeq 2500 platform. Reads were aligned to hg38 genome using HISAT2 after raw reads trimmed with Cutadapt v0.5.0. (42). Counts and fragments per kilobase of exon model per million mapped reads (FPKM) were generated using FeatureCounts (43). Raw counts were processed with DESeq2 (37)to determine differentially expressed genes. GO and KEGG analysis was performed using clusterProfiler R package (44) visualized with ggplot2 R package(45). GSEA was performed on pre-ranked gene lists according to the DESeq2 fold change, using 1,000 gene set permutations and the classic enrichment statistic. Gene sets were from gene ontology database (GO: 0006281) and MsigDB database (“HALLMARK_UNFOLDED_PROTEIN_RESPONSE", “HALLMARK_MTORC1_SIGNALING”, “GO_CELL_CYCLE_DNA_REPLICATION” ). Heatmap plots were generated using pheatmap R package(46).

**Puromycin labeling and drug treatment.** Cells were treated with DMSO, Tg, or CHX for the indicated time before labeling. Then, the media was replaced with a labeling medium containing 10 mg/ml of puromycin for 30 min(7). Cells were lysed and protein concentration was measured by bicinchoninic acid (BCA) assay. Equal amounts of protein were loaded into the gel and transferred to the membrane. Signals were detected by an anti-puromycin antibody (Sigma, MABE343). The signal from total protein loading was detected by coomassie blue staining as a control.

### Phos-tag analysis. Cell protein lysates for Phos-tag analysis were preaged as described above. Phos-tag gel was modified from our previous report (47) with the following running conditions: 15 mA for 15 min followed by 5 mA for 9.5 hours for PERK using 11.5 µM Phos-tag (APExBIO Acrylamide, F4002 ), and 100V for 3 hours for IRE1α using 75μM Phos-tag.

**Animal study.** Male C57BL/6J mice were aged 13-14 weeks were purchased from Guangdong Medical Laboratory Animal Center. All mice were maintained under a standard humidity- and temperature-controlled environment on a 12-hour light/dark cycle, with free access to food and water. The animal protocol was reviewed and approved by the Institutional Animal Use and Care Committee of Sun Yat-Sen University. For generating control mice and RBBP8-silenced mice, mice were injected with shRBBP8 adenovirus (pADV-U6-shRBBP8-CMV-EGFP, 1~4X10^9^ PFU/mouse) and control adenovirus (pDKD-CMV-eFGP-U6-shRNA, 1~4X10^9^ PFU/mouse) by tail vein injection respectively. After 14 days of adenovirus injection, for acute tunicamycin treatment, mice were injected intraperitoneally with 1mg/kg body weight of tunicamycin for 6 hours or 24 hours, and mice were sacrificed at indicated time points for subsequent analysis. All mice were randomly assigned to different treatment groups. Plasma ALT and AST levels were measured by Alanine aminotransferase Assay Kit (Nanjing Jiancheng Bioengineering Institute, C009-2-1) and Aspartate aminotransferase Assay Kit (Nanjing Jiancheng Bioengineering Institute, C010-2-1) respectively. For hepatic TG measurement, approximately 50 mg of frozen liver tissue was extracted in 1ml of saline for dissolving the lipid pellet, then TG was measured by a Triglyceride assay kit (Nanjing Jiancheng Bioengineering Institute, A110-1-1).

**TUNEL assay.** Paraffin-embedded liver sections were dewaxed, rehydrated, and incubated in permeabilizing solution with proteinase K at 37 ℃ for 30 minutes. TUNEL assay was performed using an In-Situ Cell Death detection kit (Roche, 11684795910) per the manufacturer’s protocol(41). Liver sections treated with DNase I (ThermoFisher Scientific, EN 0523) for 30 minutes at 37°C were used as positive controls. Images were obtained by a Pannoramic MIDI scanner.

**Transmission electron microscopy.** Mice after 6-hour or 24-hour tunicamycin injection were killed. The liver was immediately sliced into 1 to 2 mm^3^ pieces and fixed and embedded in Poly/bed 812 (Polysciences)(41). Fixation and embedding processes were carried out by Servicebio Inc. Embedded samples were cut with Leica Ultracut Ultramicrotome system and images were taken using JEM-1400 TEM at Sun Yat-sen University.

**Sucrose gradient ultracentrifuge analysis.** As previously reported (14), liver tissues were collected and immediately frozen in liquid nitrogen. 50mg of liver samples were lysed in 1ml lysis buffer (150mmol/L NaCl, 1mmol/L EDTA, 50mmol/L Tris, 1% TritonX-100, and 10mmol/L NEM), followed by centrifuge at 300g, 4℃ for 10min. Supernatant were centrifuged by 11ml of 10%–40% sucrose gradients (150mM NaCl, 1mM EDTA, 50mM Tris-HCl, pH 7.5), progressively layering from higher to lower density sucrose fractions in 10% increments. Extracts were ultracentrifuged at 58,000 rpm, 4℃ for 14.5h using an SW60Ti rotor (radius=120.3mm, Beckman Coulter). The 12ml-gradient was divided evenly into 6 fractions (No.1-6), and the pellet was resuspended and lysed in 1ml lysis buffer as fraction No.7. Fractions were subjected to Western blot analyses under denaturing or non-denaturing conditions.

**Human liver sample.** The HCC liver biopsies were obtained from patients with biopsy-proven as well as medical imaging-proven hepatocellular carcinoma. The normal control liver biopsies were obtained from patients without HCC who underwent surgery for excision of hepatic hemangioma in The Third Affiliated Hospital, Sun Yat-Sen University. Exclusion criteria included known acute or chronic liver disease, except for viral hepatitis, obesity or type 2 diabetes mellitus, excessive alcohol ingestion, or the use of pharmacological treatments. Patients gave written consent for their tissues to be collected. The study of these specimens was approved by the Ethics Committee of The Third Affiliated Hospital, Sun Yat-Sen University, and was conducted by the 1975 Declaration of Helsinki.

**Statistical analysis.** Results were statistically compared using the Ordinary One-way ANOVA and Two-way ANOVA followed by different multiples comparison post-tests (Tukey’s Multiple Comparison Test or Bonferroni’s Multiple Comparison Test). When pertinent, Student’s t-test was performed for unpaired or paired groups. In all plots p values are show as indicated: *, *p*<0.05, **, *p* <0.01, ***, *p* <0.001 were considered significant.

**SI References**

**References:**

1. Zhang H, Zhang Y, Zhou X, Wright S, Hyle J, Zhao L, An J, et al. Functional interrogation of HOXA9 regulome in MLLr leukemia via reporter-based CRISPR/Cas9 screen. Elife 2020;9.

2. Panganiban RA, Park HR, Sun M, Shumyatcher M, Himes BE, Lu Q. Genome-wide CRISPR screen identifies suppressors of endoplasmic reticulum stress-induced apoptosis. Proc Natl Acad Sci U S A 2019;116:13384-13393.

3. Love MI, Huber W, Anders S. Moderated estimation of fold change and dispersion for RNA-seq data with DESeq2. Genome Biol 2014;15:550.

4. Li W, Xu H, Xiao T, Cong L, Love MI, Zhang F, Irizarry RA, et al. MAGeCK enables robust identification of essential genes from genome-scale CRISPR/Cas9 knockout screens. Genome Biol 2014;15:554.

5. Qi L, Yang L, Chen H. Detecting and quantitating physiological endoplasmic reticulum stress. Methods Enzymol 2011;490:137-146.

6. Zi J, Han Q, Gu S, McGrath M, Kane S, Song C, Ge Z. Targeting NAT10 Induces Apoptosis Associated With Enhancing Endoplasmic Reticulum Stress in Acute Myeloid Leukemia Cells. Front Oncol 2020;10:598107.

7. Lafranchi L, de Boer HR, de Vries EG, Ong SE, Sartori AA, van Vugt MA. APC/C(Cdh1) controls CtIP stability during the cell cycle and in response to DNA damage. EMBO J 2014;33:2860-2879.

8. Zou T, Wang Y, Dong L, Che T, Zhao H, Yan X, Lin Z. Stabilization of SETD3 by deubiquitinase USP27 enhances cell proliferation and hepatocellular carcinoma progression. Cell Mol Life Sci 2022;79:70.

9. Bhattacharya A, Sun S, Wang H, Liu M, Long Q, Yin L, Kersten S, et al. Hepatic Sel1L-Hrd1 ER-associated degradation (ERAD) manages FGF21 levels and systemic metabolism via CREBH. EMBO J 2018;37.

10. Kim D, Paggi JM, Park C, Bennett C, Salzberg SL. Graph-based genome alignment and genotyping with HISAT2 and HISAT-genotype. Nat Biotechnol 2019;37:907-915.

11. Liao Y, Smyth GK, Shi W. featureCounts: an efficient general purpose program for assigning sequence reads to genomic features. Bioinformatics 2014;30:923-930.

12. Yu G, Wang LG, Han Y, He QY. clusterProfiler: an R package for comparing biological themes among gene clusters. OMICS 2012;16:284-287.

13. Wickham H, Sievert C. ggplot2 : Elegant graphics for data analysis. Second edition. ed. Houston, Texas: Springer, 2016: xvi, 260 pages.

14. Kolde R. pheatmap: Pretty Heatmaps. R package version 1.0.12. <https://CRAN.R-project.org/package=pheatmap>. In. ; 2019.

15. You K, Wang L, Chou CH, Liu K, Nakata T, Jaiswal A, Yao J, et al. QRICH1 dictates the outcome of ER stress through transcriptional control of proteostasis. Science 2021;371.

16. Yang L, Xue Z, He Y, Sun S, Chen H, Qi L. A Phos-tag-based approach reveals the extent of physiological endoplasmic reticulum stress. PLoS One 2010;5:e11621.

17. Sun S, Shi G, Sha H, Ji Y, Han X, Shu X, Ma H, et al. IRE1alpha is an endogenous substrate of endoplasmic-reticulum-associated degradation. Nat Cell Biol 2015;17:1546-1555.
