# Supplementary figures and images for: CRISPR screen identifies the role of RBBP8 in mediating unfolded protein response induced liver damage through regulating protein synthesis
Source: Cell Death Dis. 2023 Aug 18;14(8):531. doi: 10.1038/s41419-023-06046-x (PMC10435451; doi:10.1038/s41419-023-06046-x)

**Figure1 E**

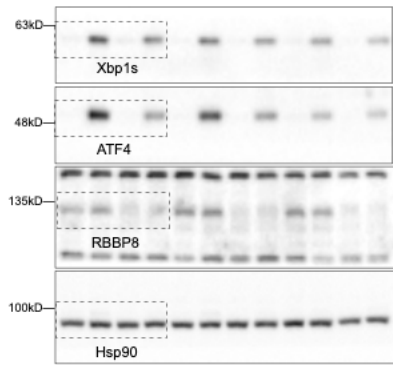

**Figure1 J**

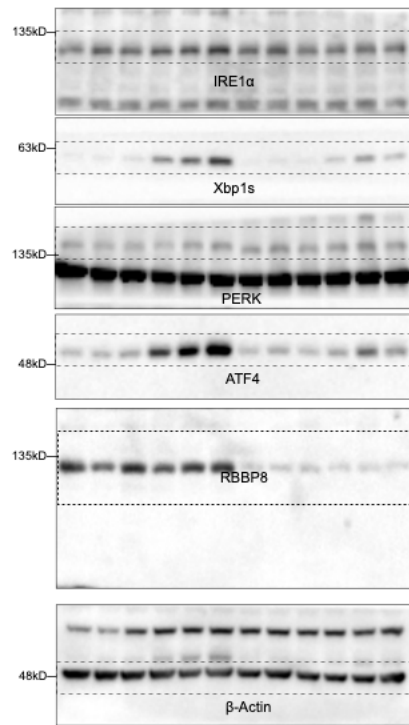

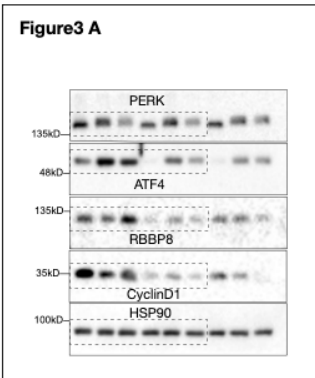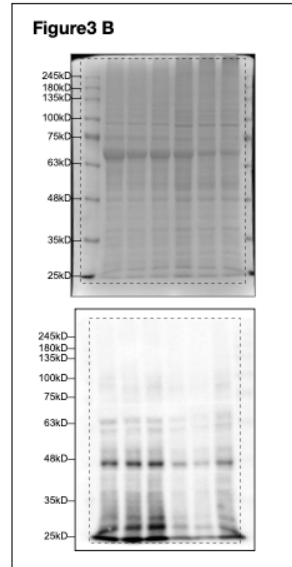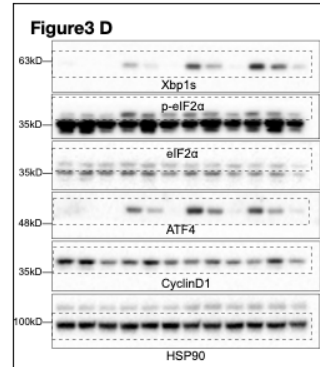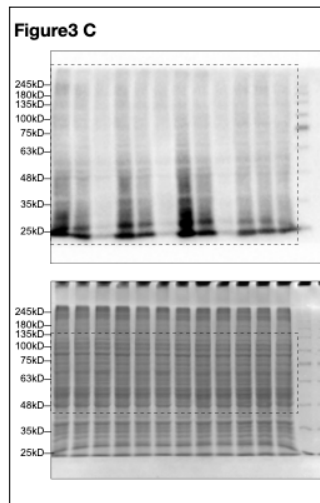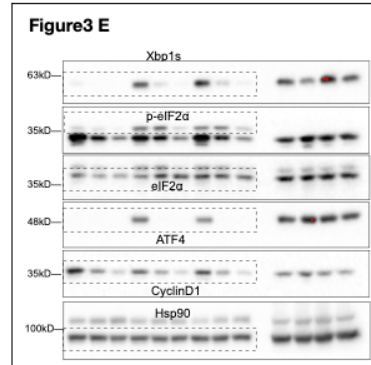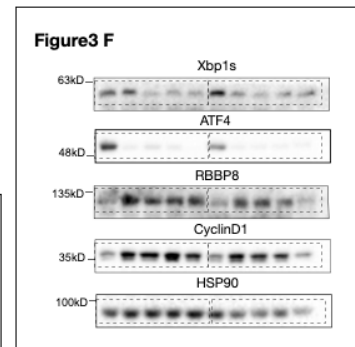

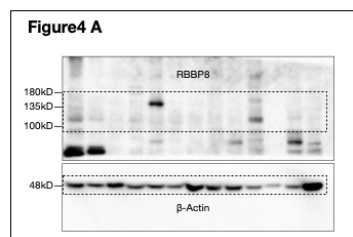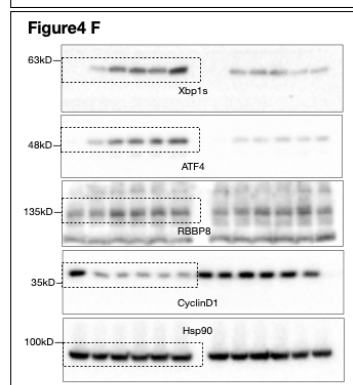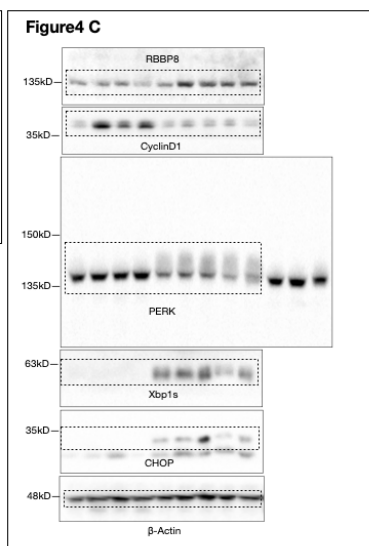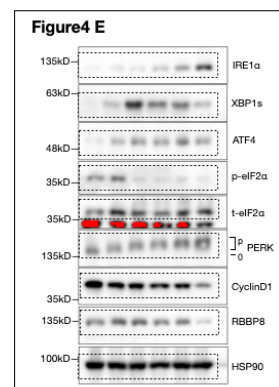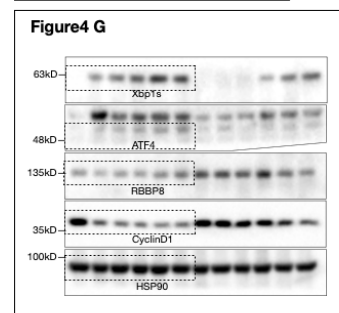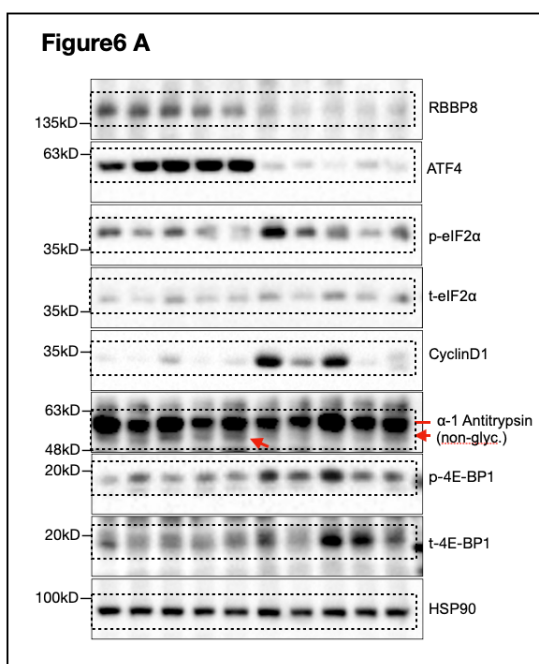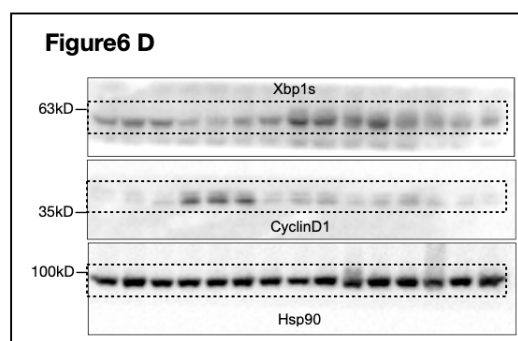

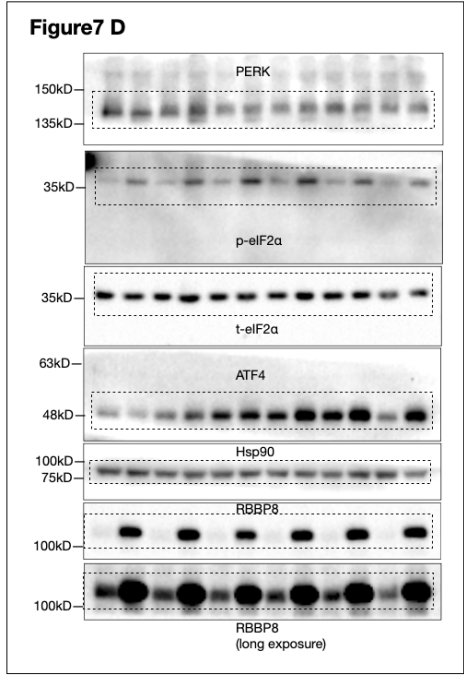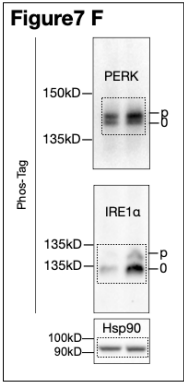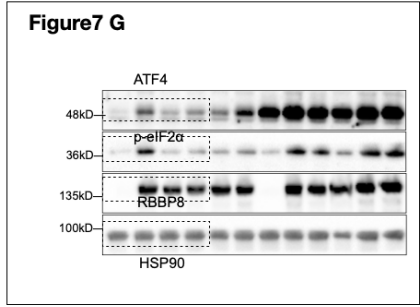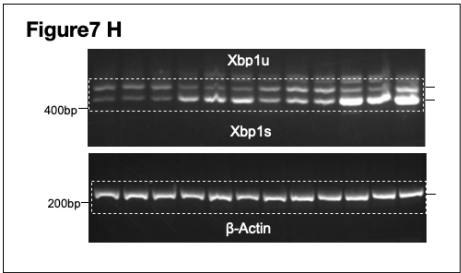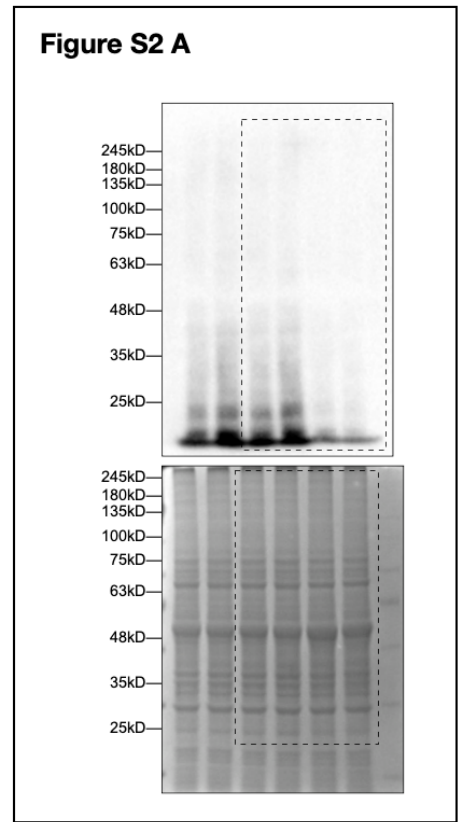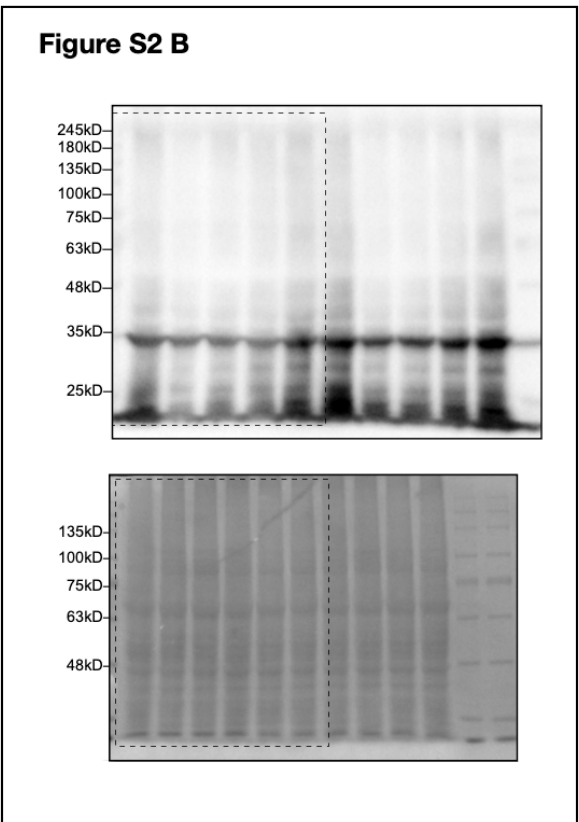

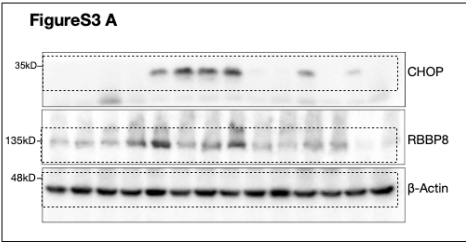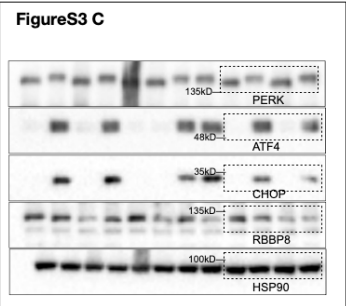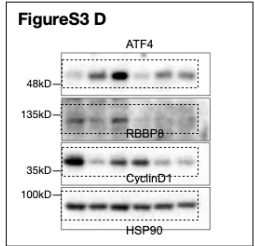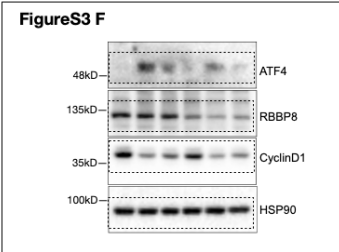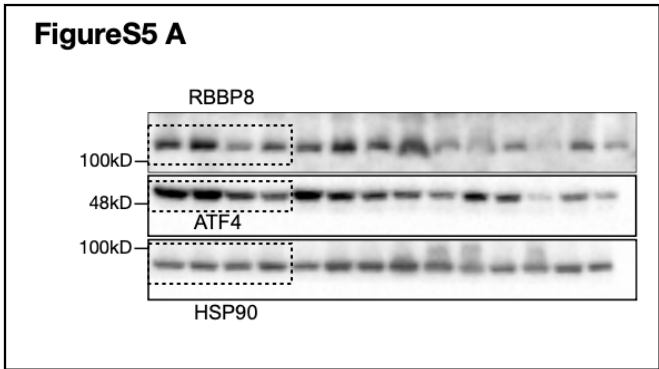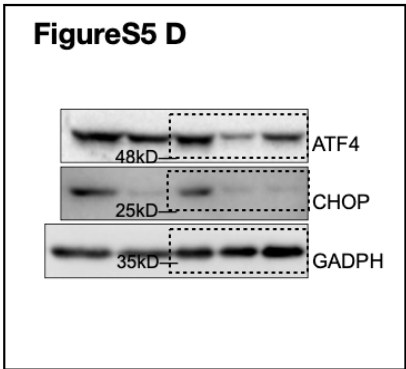

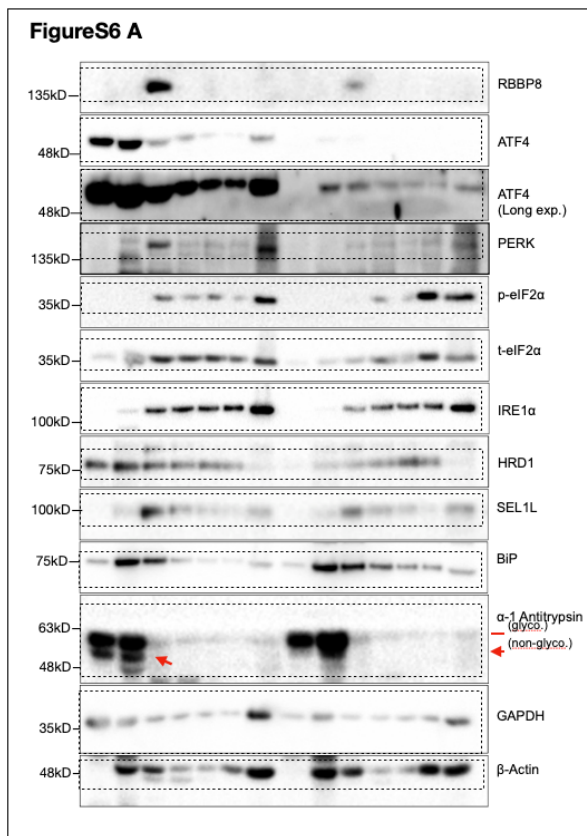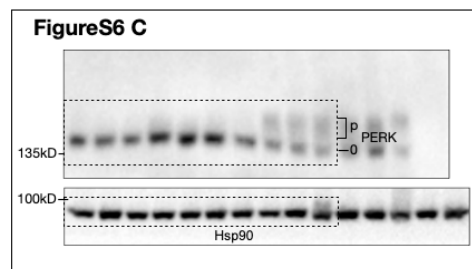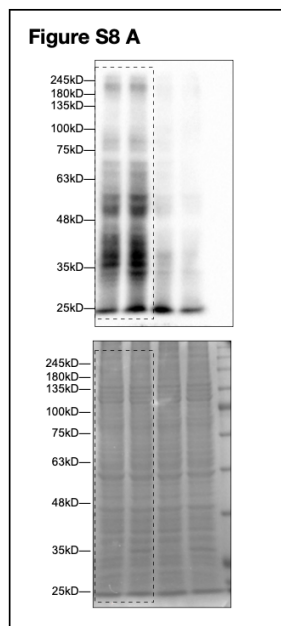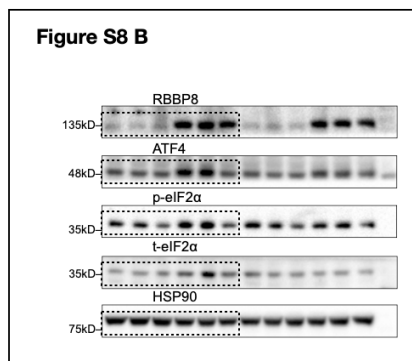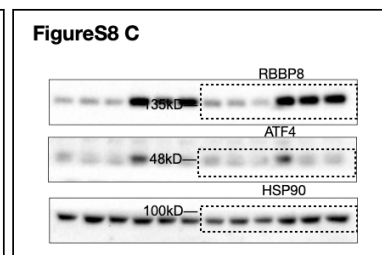

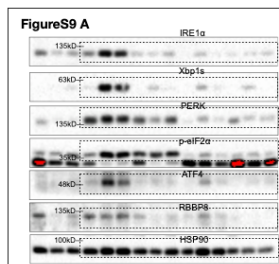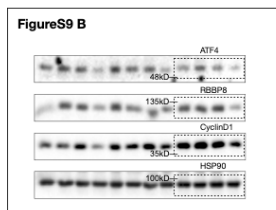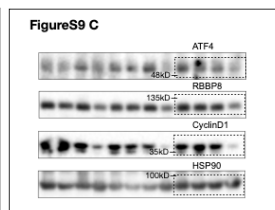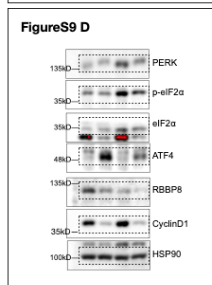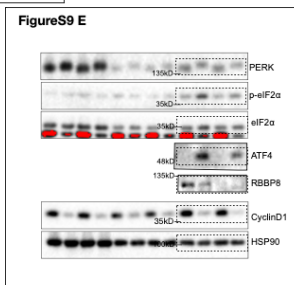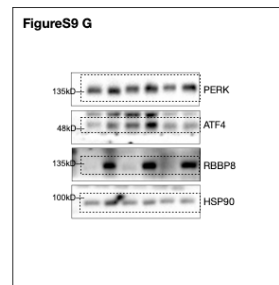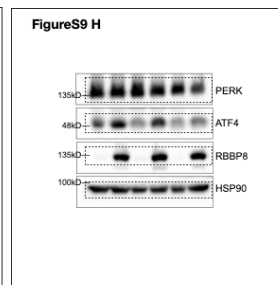

Supplement: Supplementary file 3 — Original Data File of uncrossed images [file 41419_2023_6046_MOESM3_ESM.pdf]
